# Metabolic profile and transcriptome reveal the mystery of petal blotch formation in rose

**DOI:** 10.1186/s12870-023-04057-6

**Published:** 2023-01-20

**Authors:** Naizhe Ji, Qianyu Wang, Shanshan Li, Jiaxin Wen, Liangsheng Wang, Xiaohao Ding, Shiwei Zhao, Hui Feng

**Affiliations:** 1 Beijing Key Lab of Greening Plants Breeding, Beijing Academy of Forestry and Landscape Architecture, Beijing, China; 2 Key Laboratory of Plant Resources, Institute of Botany, Chinese Academy of Sciences, Beijing, China; 3 China National Botanical Garden, Beijing, China; 4 University of Chinese Academy of Sciences, Beijing, China; 5 College of Forestry, Henan Agricultural University, Zhengzhou, China; 6 College of Food Science, Fuyang Normal University, Fuyang, China

**Keywords:** Rose, Blotch, Anthocyanin, Flavonol, Transcriptome

## Abstract

**Background:**

Petal blotch is a unique ornamental trait in angiosperm families, and blotch in rose petal is rare and has great esthetic value. However, the cause of the formation of petal blotch in rose is still unclear. The influence of key enzyme genes and regulatory genes in the pigment synthesis pathways needs to be explored and clarified.

**Results:**

In this study, the rose cultivar ‘Sunset Babylon Eyes’ with rose-red to dark red blotch at the base of petal was selected as the experimental material. The HPLC-DAD and UPLC-TQ-MS analyses indicated that only cyanidin 3,5-*O*-diglucoside (Cy3G5G) contributed to the blotch pigmentation of ‘Sunset Babylon Eyes’, and the amounts of Cy3G5G varied at different developmental stages. Only flavonols but no flavone were found in blotch and non-blotch parts. As a consequence, kaempferol and its derivatives as well as quercetin and its derivatives may act as background colors during flower developmental stages. Despite of the differences in composition, the total content of carotenoids in blotch and non-blotch parts were similar, and carotenoids may just make the petals show a brighter color. Transcriptomic data, quantitative real-time PCR and promoter sequence analyses indicated that *RC7G0058400 (F3’H)*, *RC6G0470600 (DFR)* and *RC7G0212200 (ANS)* may be the key enzyme genes for the early formation and color deepening of blotch at later stages. As for two transcription factor, RC7G0019000 (MYB) and RC1G0363600 (WRKY) may bind to the promoters of critical enzyme genes, or RC1G0363600 (WRKY) may bind to the promoter of *RC7G0019000 (MYB)* to activate the anthocyanin accumulation in blotch parts of ‘Sunset Babylon Eyes’.

**Conclusions:**

Our findings provide a theoretical basis for the understanding of the chemical and molecular mechanism for the formation of petal blotch in rose.

**Supplementary Information:**

The online version contains supplementary material available at 10.1186/s12870-023-04057-6.

## Background

Petal color patterning, such as flower spots and stripes, is one of the most significantly biological and ornamental characteristics for plants and has great significance in plant evolutionary biology [1–4]. As one of the specific floral patterns, petal blotch is found in angiosperm families, as observed for example in Cistaceae (e.g., *Cistus purpureus*), Paeoniaceae (e.g., *Paeonia rockii*), Onagraceae (e.g., *Clarkia gracilis*), Violaceae (e.g., *Viola* × *wittrockiana* Gams.) and Compositae (e.g., *Senecio cruentus*) [5–9]. Although it is sometimes associated with elaborated epidermal cell morphologies, color patterning is mainly generated by pigment accumulation in the different parts of flower petals [3, 10, 11]. Among four major classes of pigments (flavonoids, carotenoids, chlorophylls and betaines), flavonoids and carotenoids are widely participated in the color formation in most flowers [12].

Flavonoids include flavones, flavonols, anthocyanins and other compounds. Flavonoids are synthesized by a branched pathway that yields both colorless compounds (e.g. flavonols) and colored pigments (e.g. anthocyanins) [13]. As major pigmented flavonoids, anthocyanins, which cause pink, orange, red, scarlet, purple, blue and cyanic flower coloration, play a vital role in flower color development. Anthocyanin aglycones are divided into six common anthocyanidins, namely cyanidin, delphinidin, pelargonidin, peonidin, petunidin, and malvidin [14–16]. Flavonols and flavones, as colorless flavonoids, play important roles in coloration by co-pigmentation effects with anthocyanins in floral organs [17]. Carotenoid is the generic term for carotenes and xanthophylls, which provide colors ranging from yellow to orange in ornamentals [12, 14].

Pigment (anthocyanin, flavonol and carotenoid) pathways and genes have been extensively characterized in model and non-model plants [12, 18–21]. It was reported that the synthesis of anthocyanin and flavonol shares the same upstream pathway as the formation of dihydrokaempferol and dihydroquercetin, followed by downstream branch for the formation of anthocyanins and flavonols. In this comprehensive synthesis process, the key enzymes have been well characterized, including chalcone synthase (CHS), chalcone isomerase (CHI), flavanone 3-hydroxylase (F3H), flavonoid 3′-hydroxylase (F3′H), flavonoid 3′,5′-hydroxylase (F3′5′H), flavonol synthase (FLS), dihydroflavonol 4-reductase (DFR), anthocyanidin synthase (ANS) and UDP-flavonoid glucosyltransferase (UFGT) [12, 22]. The carotenoid biosynthesis pathway involves multiple enzymes like phytoene synthase (PSY), phytoene desaturase (PDS), ζ-carotene desaturase (ZDS), carotenoid isomerase (CRTISO), ε-ring cyclase (LCYE) and β-ring cyclase (LCYB) contribute to the synthesis of carotenes [12, 14]. Carotenes are catalyzed to produce various xanthophylls by enzymes such as ε-ring hydroxylase (CHYE), β-ring hydroxylase (CHYB), zeaxanthin epoxidase (ZEP), violaxanthin de-epoxidase (VDE) and neoxanthin synthase (NSY) [23].

Regulation of flavonoid pathways is mostly coordinated by a transcription factor complex consisting of R2R3 MYB transcription factors (TFs), basic helix–loop–helix (bHLH) TFs and WD40 proteins (MBW complex), which activates transcription biosynthetic genes in many plants, such as *Arabidopsis thaliana*, *Petunia hybrida* and *Rosa hybrida* [13, 24–26]. MBW complex also functions in carotenoid biosynthesis in *Medicago truncatula* [27]. Besides MYB, bHLH and WD40, many transcription factors are implicated in the control of the flavonoid biosynthetic pathway. For example, WRKY transcription factors play a role as a flavonoid regulator in Petunia [28]. NAC transcription factors are proved to be the necessary partner TFs for the anthocyanin biosynthesis in blood-fleshed peach [29]. DOF transcription factors have the negative influence on flavonoid biosynthesis in *Arabidopsis thaliana* [30].

**Fig. 1:**
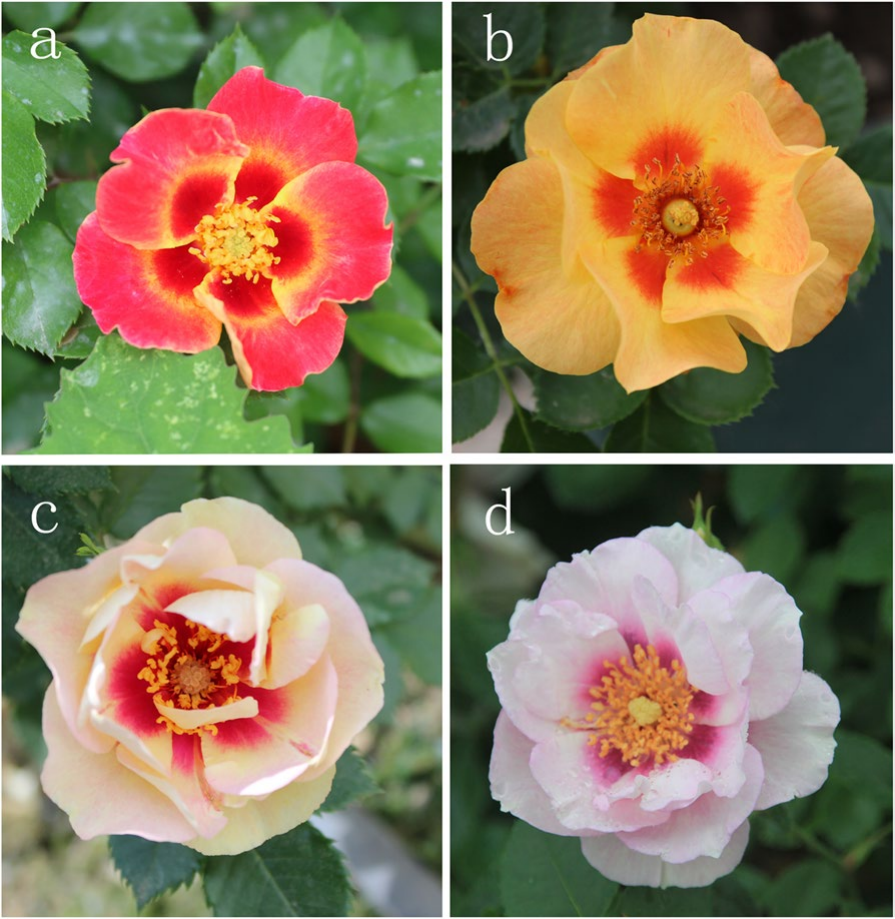
Variable Hulthemia hybrids. **a** *Rosa* ‘Sunshine Babylon Eyes ‘. **b** *Rosa* ‘Xizixiawu’. **c** *Rosa* ‘Bull’s Eye ‘. **d** *Rosa* ‘Eyes for you ‘

Roses are among the most commonly cultivated ornamental plants worldwide and have gained the title of the world’s favorite flower [31]. The genus *Rosa* contains approximately 200 species and over 40,000 cultivars [32]. *R. persica* stands out from all wild roses by the chestnut red blotch at the base of each yellow petal [33, 34]. Since Jack L. Harkness took up the persica line of breeding in 1960s and introduced one of Hulthemia hybrids in 1985 under the name ‘Tigris’, the success of hulthemia breeding has been a breakthrough in rose breeding in recent years [35]. Hulthemia hybrids with variable colors provide a good mode for studying pigment biosynthesis and pattern formation (Fig. 1). Previous studies report that rose petals are known to contain anthocyanins based on cyanidin, pelargonidin and peonidin as well as carotenoids. Roses do not produce flavones whereas two predominant flavonol aglycones, namely kaempferol and quercetin, exist in rose petals [36–41]. Yellow roses have been used for research on carotenoids in rose petals [42, 43]. Admittedly, there are various reports on rose petal coloration [44–46]. Nevertheless, the mechanism for pigmentation patterning of hulthemia hybrids has never been reported.

In this study, the hybrid hulthemia cultivar ‘Sunset Babylon Eyes’ was used to explore the pigmentation regulatory network of blotch and non-blotch parts during the development of rose flowers. We reported the metabolic profiling of flavonoids and carotenoids, as well as gene expression dynamics for the blotch and non-blotch petals of five different coloring stages using integrated analysis of the metabolome and transcriptome. With this extensive analysis of multiple approaches in hybrid hulthemia cultivar ‘Sunset Babylon Eyes’, we reveal changes in the key pigments and related biosynthesis genes that are associated with petal blotch formation.

## Results

### Flower colors of ‘sunset Babylon eyes’ at different developmental stages

**Fig. 2:**
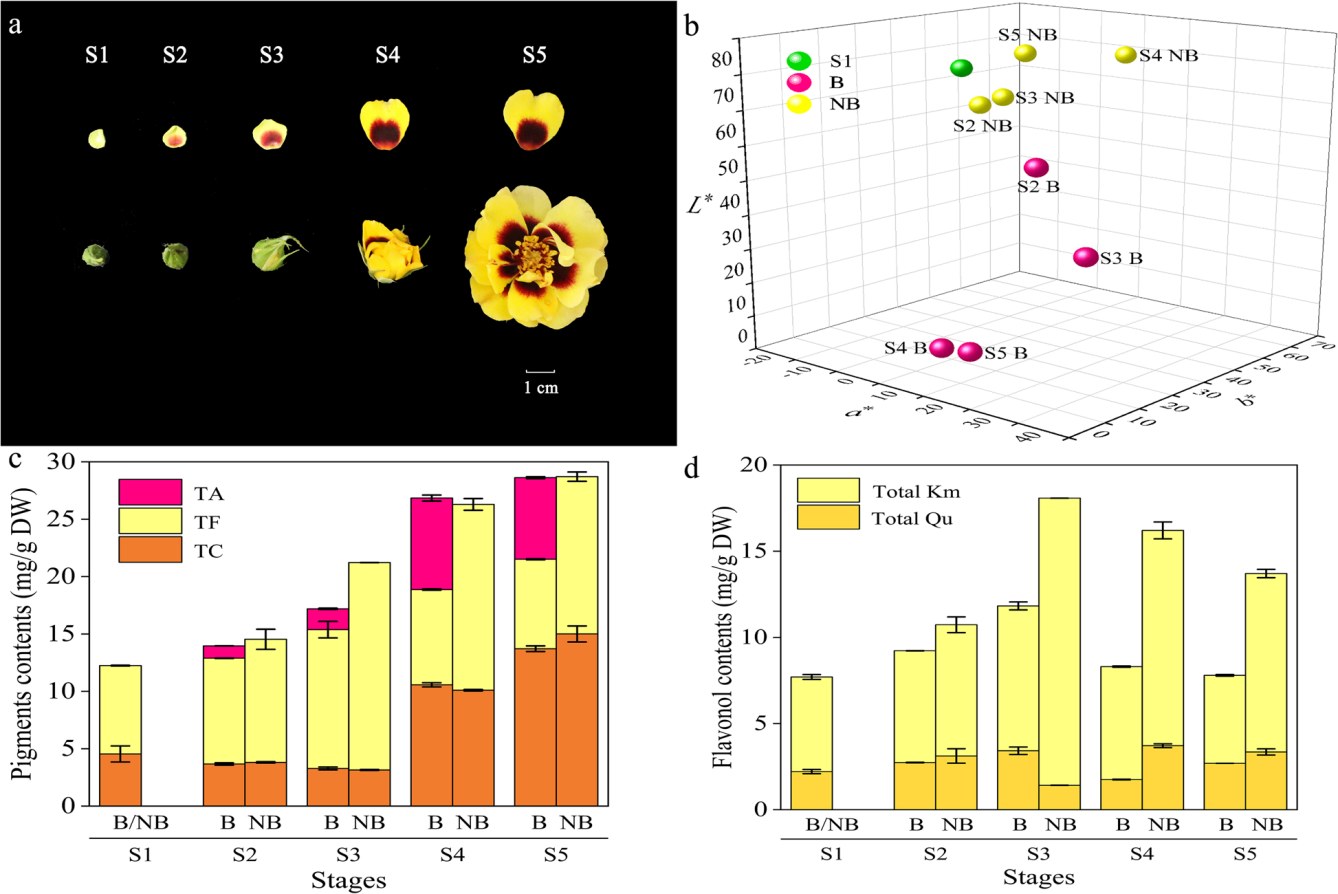
Petal blotch development and pigments accumulation in rose. **a** Phenotypes of different developmental stages of rose petal blotch. **b** Flower color distribution of *Rosa* ‘Sunset Babylon Eyes’. **c** Pigment accumulation in rose petal, reflecting the total content of anthocyanins (TA), carotenoids (TC), flavone and flavonol (TF) in the petal blotch and non-blotch regions during different developmental stages. **d** Flavonol accumulation in rose petal, including kaempferol (Total Km) and quercetin (Total Qu). Three independent biological experiments were performed. Values represent means ± SE

We collected materials at five stages depending on the progress of anthesis and development of blotch [8, 47, 48]. At S1 (colorless bud petal stage), petals were yellow-green without blotches. Cerise blotches appeared at the base of the light yellow petals at S2 (initially colored bud petal stage). At S3 (colored bud petal stage), blotches grew to about half of the whole petal and turned rose-red while non-blotch parts became butter yellow. Blotches grew continuously and their color turned crimson while the non-blotch parts became bright yellow at S4 (initiating blooming stage). At S5 (blooming stage), non-blotch parts were yellow while blotches turned dark red (Fig. 2a).

To precisely evaluate the color of rose flowers, color parameters L^*^, a^*^ and b^*^ of CIEL^*^a^*^b^*^ color system of petals were measured. Significant differences were observed in color parameters among the blotch and non-blotch parts at different stages (Fig. 2b). L^*^ value of blotch and non-blotch parts declined from S1 to S2. From S2 to S5, L^*^ value of the non-blotch parts increased whilst that of the blotch parts decreased. Parameter a^*^ represents green and red color from negative value to positive value. The a^*^ value of non-blotch parts saw a slight increase from S1 to S4, and then declined from S4 to S5. The a^*^ value of blotch parts witnessed a fluctuation trend of ‘increase-decrease-increase’ from S1 to S5 and stood at the peak at S3. Parameter b^*^ represents blue and yellow color from negative value to positive value. The b^*^ value of the non-blotch parts increased continuously from S1 to S4 and then dropped substantially. Conversely, the b^*^ value of the blotch parts experienced an opposite trend.

### Identification and quantification of flavonoids in petals of ‘sunset Babylon eyes’

Only one anthocyanin: cyanidin 3,5-*O*-diglucoside (Cy3G5G) was found in the blotch parts from S2 to S5 (Table 1, Supplementary Fig. 1a, Supplementary Table S1). Anthocyanins were not detected at S1 and in the non-blotch parts from S2 to S5. The content of Cy3G5G was very low in the blotch parts at both of S2 and S3. And the total content of anthocyanin (TA) in the blotch part at S4 was the highest, which was about 7.4 times higher than that of the blotch parts at S2. TA in the blotch part at S5 was close to but slightly lower than that at S4 (Fig. 2c).

**Table 1:** Identification of flavonoids

Peak no.	Retention time (min)	λ_**vis-max**_ (nm)	λ_**vis-acyl**_ (nm)	ESI^**−**^MS^**−**^(***m/z***)	Aglycone	Main identified molecule	References	Standard
a1	15.708	511	277	611, 449, 287	Cyanidin	Cyanidin 3,5-*O*-diglucoside	/	Cyanidin 3,5-*O*-diglucoside
f1	28.061	328	257	433, 301	Quercetin	Quercetin 3-*O*-α-L-arabinofuranoside	[39, 41, 49]	/
f2	29.021	340	257	593, 431, 285	Kaempferol	Kaempferol 3-*O*-rhamnosyl-7-*O*-glucoside	[38]	Kaempferol 3-*O*-rhamnoside-7-*O*-glucoside
f3	29.984	359	254	609, 447, 301	Quercetin	Quercetin 3-*O*-rutinoside	[38]	Quercetin 3-*O*-rutinoside
f4	30.328	347	265	447, 285	Kaempferol	Kaempferol 3-*O*-glucoside	[38, 41]	Kaempferol 3-*O*-glucoside
f5	30.816	347	256	447, 301	Quercetin	Quercetin 3-*O*-rhamnoside	[49]	Quercetin 3-*O*-rhamnoside
f6	31.118	360	257	463, 301	Quercetin	Quercetin 3-*O*-galactoside	[38, 41, 49]	Quercetin 3-*O*-galactoside
f7	31.621	347	257	599, 447, 285	Kaempferol	Kaempferol 3-*O*-(galloyl)-glucuronide or kaempferol 7-*O*-(galloyl)-glucuronide	[42]	/
f8	32.164	359	265	447, 285	Kaempferol	Kaempferol 7-*O*-glucoside	[38, 41]	Kaempferol 7-*O*-glucoside
f9	33.168	347	265	417, 285	Kaempferol	Kaempferol 3-*O*-arabinoside	[39, 49]	Kaempferol 3-*O*-arabinoside
f10	33.734	304	265	431, 285	Kaempferol	Kaempferol 3-*O*-α-D-rhamnoside	[38, 39, 41, 49]	Kaempferol 3-*O*-α-D-rhamnoside
f11	46.593	/	/	285	Kaempferol	Kaempferol	[42]	Kaempferol

Eleven flavonol glycosides and no flavone glycosides were identified (Supplementary Fig. 1b; Table 1). Kaempferol (f11) was solely detected at S1. Meanwhile, kaempferol 3-*O*-α-D-rhamnoside (f10) was not detected at S1 and S2, but the compound began to accumulate at S3. Among the flavonol glycosides, f7, which was characterized as kaempferol 3 -*O*-(galloyl)-glucuronide or kaempferol 7 -*O*-(galloyl)-glucuronide, was the predominant component in the blotch parts at S1, S2 and S3, but no longer accumulated at S5. However, f8 (kaempferol 7 -*O*-glucoside) and f10 (kaempferol 3 -*O*-α-D-rhamnoside) were the predominant flavonols in non-blotch parts at S3, S4 and S5 (Supplementary Table S1). Therefore, kaempferol glycosides may play an important role in the coloration of the non-spot parts of ‘Sunset Babylon Eyes’. At S1 and S2, f7 (kaempferol 3-*O*-(galloyl)-glucuronide or kaempferol 7-*O*-(galloyl)-glucuronide) was the key compound for petals to show yellow. From S3 to S5, f8 (kaempferol 7 -*O*-glucoside) played a major role in coloration.

The total content of flavones and flavonols (TF) at S5 was the lowest, followed by that at S1. TF in the non-blotch parts were always higher than that in the blotch parts, peaking at S3 in the non-blotch part (Fig. 2c). The content of total kaempferol and its derivatives (Total Km) was always higher than that of total quercetin and its derivatives (Total Qu) at all surveyed stages. From S1 to S3, Total Km and Total Qu increased. The content of total flavones and flavonols (TF) remained stable between S4 and S5, but Total Qu at S5 was higher than that at S4. Total Qu of the non-blotch parts reached the lowest point at S3, whilst there was little difference in other stages. Total Km increased gradually from S1 to S3, and reached the peak at S3. From this point onward, total Km declined steadily from S3 to S5 (Fig. 2d). Consequently, the accumulation of anthocyanin resulted in the formation and deepening of blotch, whereas the yellow non-blotch parts of ‘Sunset Babylon Eyes’ may be related to the synthesis of flavonols.

### Identification and quantification of carotenoids in petals of ‘sunset Babylon eyes’

Twelve carotenoids were detected and identified (Supplementary Table S1, Supplementary Table S2). At S1, S2 and S3, petals contained only two carotenoids: (all-*E*)-Lutein (c7) and (all-*E*) -β-Carotene (c12). It is worthwhile mentioning that the content of c7 was always higher than that of c12. At S4 and S5, the content of c7 decreased and the distribution of each component was relatively average except c12. The total content of carotenoids (TC) at S1 were slightly higher than that at S2 and S3. The categories and the total content of carotenoids began to surge from S4, reaching the maximum at S5. At S4 and S5, blotch parts differed from non-blotch parts in carotenoid compositions (Supplementary Table S1). The types and total content of carotenoids reached the peak in the non-blotch part at S5. Collectively, TC experienced a similar trend as the brightness of petals (L^*^) (Fig. 2c), and hence carotenoids may mainly have impact on the brightness of ‘Sunset Babylon Eyes’ petals.

### Transcriptome analysis of ‘Sunset Babylon Eyes’

To study the molecular basis of petal blotch formation in rose, library preparation and RNA-seq were performed on non-blotch and blotch parts at five stages. After filtering these raw reads, the clean reads were obtained, which ranged from 36,716,138 to 43,862,630 (Supplementary Table S3). The Q30 value of all 27 libraries were more than 91%. Between 81.28 and 83.30% of the sequenced reads could be aligned to the rose reference genome (Supplementary Table S3).

### Comparisons of the DEGs between non-blotch and blotch parts at different developmental stages

To analyze the DEGs between non-blotch and blotch parts at different developmental stages, comparisons were conducted between six groups. The DEGs were filtrated according to an expression level |log2(FC) | > 1 and *p*-value < 0.05 in each pairwise comparison. Upregulated DEGs and downregulated DEGs were counted, as displayed in Supplementary Fig. 2. The number of DEGs was much higher at the S1vs S2B and S1vs S2NB comparisons than other four comparisons, indicating S2, which was the time the petal blotch appeared, was a vital stage for the formation of petal blotch. From S2 to S5, the number of DEGs dropped to the lowest point at the S3NB vs. S3B comparison (508 upregulated/downregulated genes) and reached a peak at the S4NB vs. S4B comparison (1855 upregulated/downregulated genes), illustrating that there are dramatic changes between non-blotch and blotch parts at S4. Anthocyanin different accumulation was the main cause of blotch formation, while all DEGs (9 upregulated/4 downregulated genes), which were found in S1vs. S2B, S2NB vs. S2B, S3NB vs. S3B, S4NB vs. S4B and S5NB vs. S5B datasets (Supplementary Fig. 3) were not related to anthocyanin synthesis pathway. These results indicated that different genes affected the anthocyanin accumulation at different petal developmental stages.

### GO functional enrichment and KEGG pathway enrichment analysis of DEGs

We analyzed the GO and KEGG pathways to determine the biological functions of DEGs. 16,367 (41.26%) and 13,695 (34.52%) genes were annotated to GO and KEGG databases. The GO annotation system consisted of three major branches: biological process, molecular function, and cellular component. Phosphotransferase activity, cinnamyl−alcohol dehydrogenase activity, xyloglucosyl transferase activity, sequence−specific DNA binding, secondary active sulfate transmembrane transporter, and isoleucine−tRNA ligase activity were the most significant enrichment GO terms under the molecular function category among six comparisons, respectively. Meanwhile, under the biological process category, DNA packaging, defense response, xyloglucan metabolic process, auxin metabolic process, sulfate transport, and response to biotic stimulus were the most significant enrichment GO terms among six comparisons, respectively (Supplementary Fig. 4).

In the KEGG signal enrichment pathway, phenylpropanoid pathway and carotenoid biosynthesis were enriched at each comparison. Phenylpropanoid pathway was the upstream pathway of flavonoid biosynthesis and anthocyanin synthesis. In the S2NB vs. S2B, S3NB vs. S3B and S4NB vs. S4B comparisons, anthocyanin synthesis pathway was enriched. Meanwhile, flavonoid biosynthesis pathway was enriched in S2NB vs. S2B and S5NB vs. S5B comparisons (Supplementary Fig. 5).

### Mining of enzyme genes in the transcriptome of ‘sunset Babylon eyes’

**Fig. 3:**
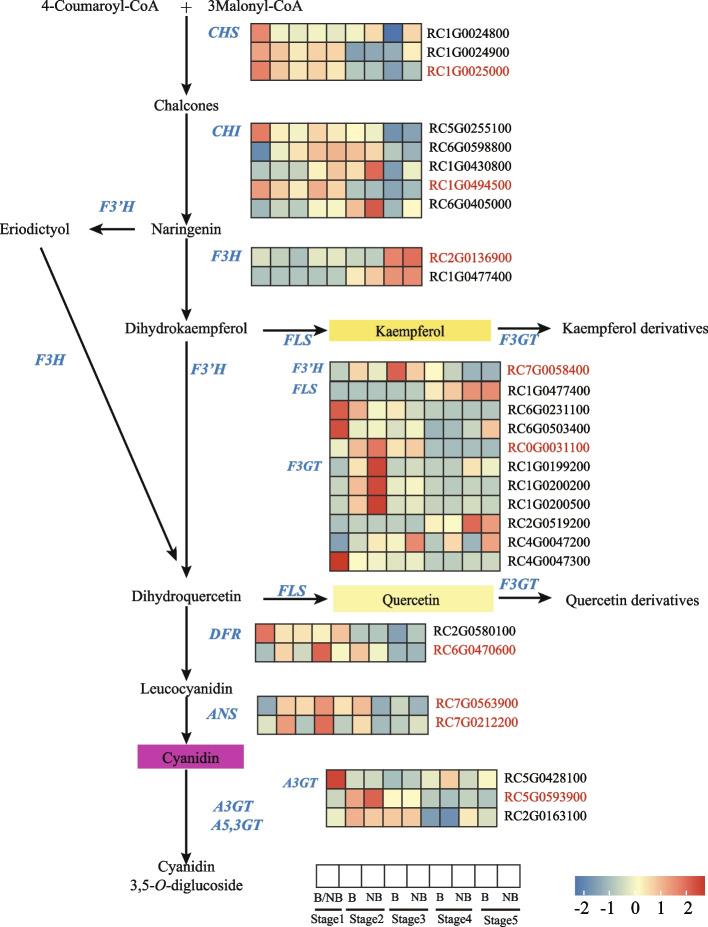
Differential expression of unigenes and metabolites related to the flavonoid metabolism pathway. B: the blotch part of the petal. NB: the non-blotch part of the petal. Genes marked red were candidate genes

To investigate the pathways of pigment synthesis in the blotch and non-blotch parts of petals, we analyzed the genes involved in anthocyanin and flavonol biosynthesis pathways in *Rosa* ‘Sunset Babylon Eyes’. After searching all these genes in the functional annotations, a total of 143 different expression genes involved in the above pathways (Table 2) were found. 28 of 143 genes were focused on because the expression levels of the 28 key enzyme genes saw the similar trends with the content of anthocyanin or flavonol. The chosen enzyme genes contained upstream genes (*CHS, CHI*, etc.) and downstream genes (*DFR, ANS*, etc.) (Fig. 3, Table 2). As the upstream genes of anthocyanin and flavonol biosynthesis pathways, the majority of *CHS, CHI* and *F3H* expressed higher at early stages (Fig. 3). Among them, two enzyme genes, *RC1G0025000* (*CHS*) and *RC1G0494500* (*CHI*), both had the highest expression level at S1 and expressed higher in blotch parts from S2 to S3. *RC2G0136900* (*F3H*) has the highest expression level at S5, except that the expression level in the blotch part at S2 was higher, from S3 to S5, its expression in the non-blotch parts was lower than or equal to that in the blotch parts.

**Table 2:** Candidate unigenes involved in anthocyanin and flavonol biosynthesis in rose

Function	Gene name	Gene abbrevia-tion	Total No.	DEG No.
Flavonoid synthesis pathway	Chalcone synthase	*CHS*	7	3
	Chalcone isomerase	*CHI*	7	5
	Flavanone 3-hydroxylase	*F3H*	4	2
	Flavonoid 3′-hydroxylase	*F3’H*	15	1
	Flavonoid 3′,5′-hydroxylase	*F3’5’H*	5	0
	Dihydroflavonol 4-reductase	*DFR*	6	2
Anthocyanin synthesis pathway	Anthocyanidin synthase	*ANS*	7	2
	UDP-glucose: anthocyanin 3-glucosylltransferase	*A3GT*	11	3
	Anthocyanidin 3-*O*-glucoside 2″-*O*-glucosyltransferase Anthocyanidin 3-*O*-glucoside 6″-*O*-glucosyltransferase	*A3GGT*	7	0
	Anthocyanidin 5, 3-*O*-glucosyltransferase	*A5, 3GT*	3	0
Flavone and flavonol biosynthesis pathway	flavonoid 3-glucosyltransferase	*F3GT*	4	0
	UDP-glucose flavonoid 3-*O*-glucosyltransferase		52	6
	UDP-glucose: flavonol 3-glucosyltransferase		3	0
	Flavonol synthase	*FLS*	12	4
Transcription factors	AP2/B3 transcription factor family protein	*AP2*	13	1
	basic helix-loop-helix (bHLH) DNA-binding superfamily protein	*bHLH*	63	7
	Dof-type zinc finger DNA-binding family protein	*DOF*	13	3
	myb domain protein	*MYB*	85	9
	NAC domain containing protein	*NAC*	119	8
	TCP domain protein	*TCP*	9	3
	Transducin/WD40 repeat-like superfamily protein	*WD40*	235	6
	WRKY DNA-binding protein	*WRKY*	55	6

*FLS* and *UF3GT* were key enzyme genes for the biosynthesis and modification of flavonol compounds, thus genes which expressed higher in the non-blotch parts from S1 to S3 were got more attention. Among them, the expression level of *RC0G0031100* (*FLS*) was the highest in the non-blotch part at S2. In addition, *RC5G0593900* (UDP-glucose: anthocyanin 3-glucosylltransferase, *A3GT*), whose expression level was higher in blotch parts from S1 to S3, could also be the key gene for the modification of flavonol compounds. As the downstream genes of anthocyanin synthesis pathway, the majority of *F3’H*, *DFR*, and *ANS* genes expressed higher in the blotch parts from S2 to S4.

In summary, *CHS* and *CHI* were consistent with the formation and accumulation of anthocyanin and flavonol. *F3’H, DFR* and *ANS* were related to the accumulation of Cy3G5G and the formation of the dark-red blotch, whereas *FLS* and *A3GT* were involved in the accumulation of flavonol compounds in non-blotch parts.

### Mining of regulatory genes in the transcriptome of ‘Sunset Babylon Eyes’

**Fig. 4:**
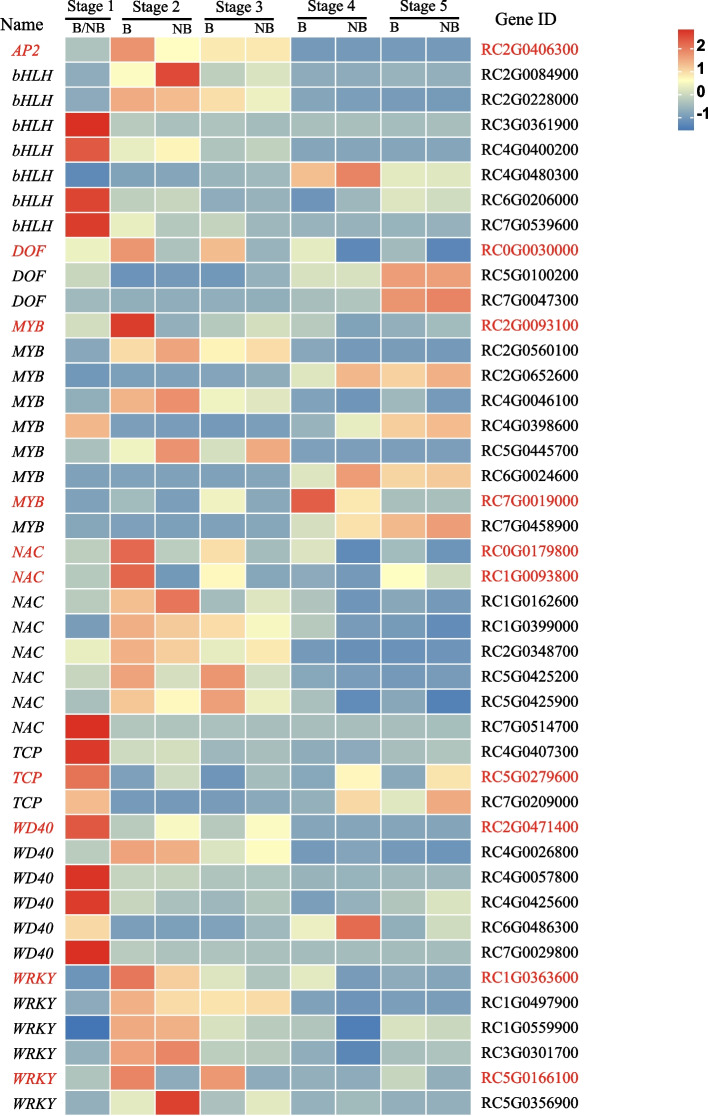
Differential expression of transcription factors related to the flavonoid metabolism pathway. B: the blotch part of the petal. NB: the non-blotch part of the petal. Genes marked red were candidate genes

Some transcription factor (TF) families including MYB, bHLH, AP2, DOF, NAC, TCP, WD40 and WRKY play important roles in color formation via anthocyanins biosynthesis by regulating the expression of key enzyme genes. We therefore analyzed the expression pattern of 592 genes belonging to these TF families in blotch and non-blotch parts (Table 2) and 43 DEGs were found (Fig. 4). Of 43 genes, there were 13 *DOF*, 9 *MYB*,8 *NAC*,7 *bHLH*, *6 WD40*, 6 *WRKY*, 3 *TCP* and 1 *AP2*.

Among them, *RC5G0279600 (TCP)* and *RC2G0471400 (WD40)* had the highest expression level at S1 and the expression level in all the blotch parts were lower than or equal to those in the non-blotch parts. The color change of blotch and non-blotch parts of ‘Sunset Babylon Eyes’ may be related to the decrease of the expression of these two regulatory genes. *RC7G0019000* (*MYB*) was an up-regulated gene in blotch parts and expressed highest at S4. However, as up-regulated genes in blotch parts, *RC1G0363600 (WRKY), RC2G0406300 (AP2), RC5G0166100 (WRKY), RC1G0093800 (NAC), RC2G0093100 (MYB), RC0G0030000 (DOF)*, and *RC0G0179800 (NAC)* expressed at the highest level in the blotch part at S2. Seven *bHLH* expressed the highest at S1 or in the non-blotch parts, and thus they may not be the key transcription factors with direct effect on the blotch pigmentation.

Collectively, *RC5G0279600* (*TCP*) and *RC2G0471400* (*WD40*) may be negative regulatory genes for blotch formation. *RC7G0019000* (*MYB*), *RC1G0363600* (*WRKY*), *RC2G0406300* (*AP2*), *RC5G0166100* (*WRKY*), *RC1G0093800* (*NAC*), *RC2G0093100* (*MYB*), *RC0G0030000* (*DOF*), and *RC0G0179800* (*NAC*) may be positive regulatory genes for blotch formation.

### Quantitative real-time PCR (qRT-PCR) analysis of candidate enzyme genes and regulatory genes

**Fig. 5:**
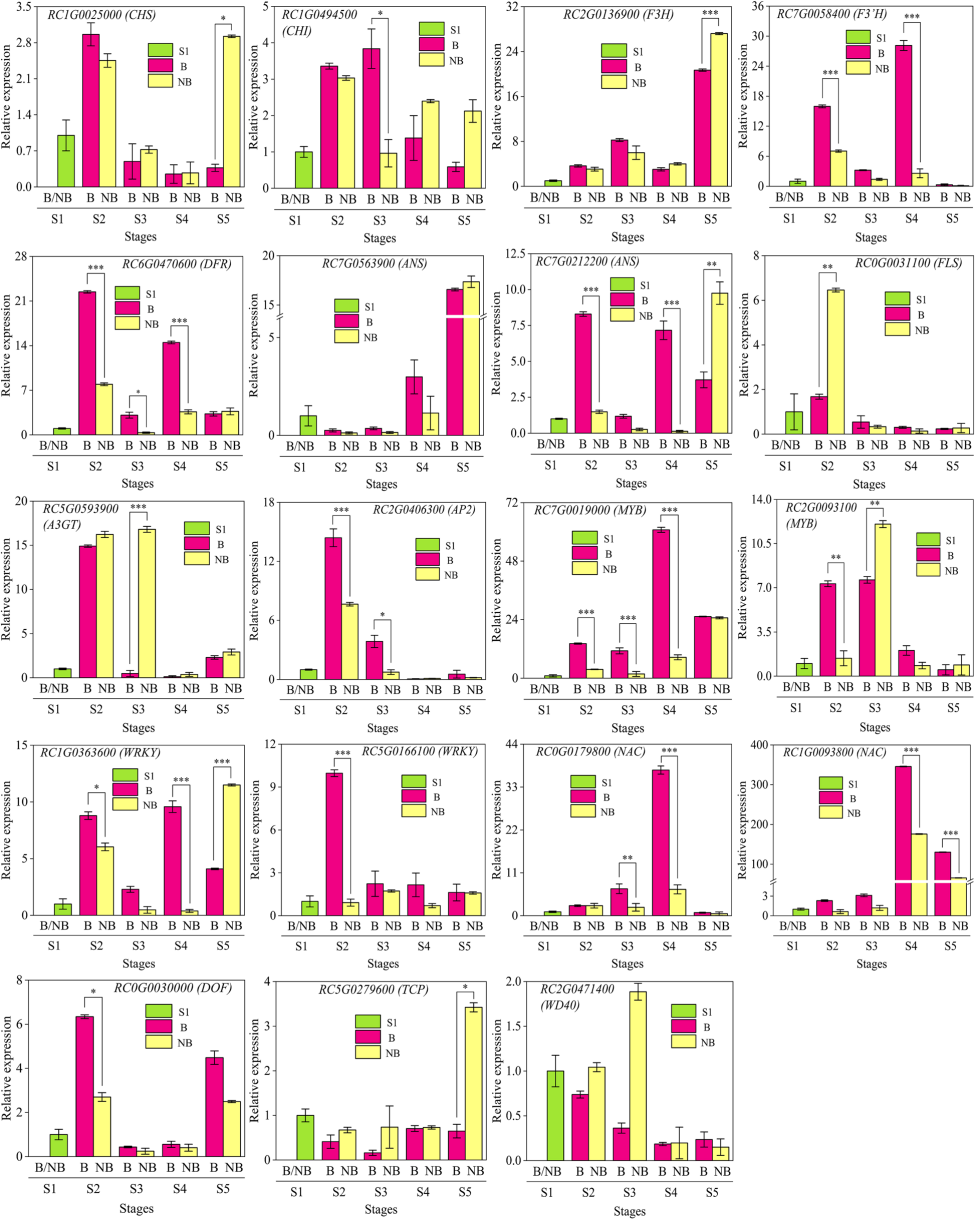
Relative expression level of candidate genes by qRT-PCR. Three independent biological experiments were performed. Values represent means ± SE (*p < 0.05; **p < 0.01; ***p < 0.001). B: the blotch part of the petal. NB: the non-blotch part of the petal

Based on the principle that the expression of enzyme genes was related to the accumulation of anthocyanin and flavonol, 9 candidate enzyme genes were chosen for qRT-PCR. Depending on the principle of the same or opposite expression of enzyme genes, qRT-PCR analysis was performed on 10 candidate regulatory genes. It was found that the fitting rate of expression trends with transcriptome data in each stage reached 89.47%. According to the qRT-PCR results, *RC1G0025000 (CHS)*, *RC1G0494500 (CHI)*, *RC2G0136900 (F3H)* may be involved in the initiation of ‘Sunset Babylon Eyes’ blotch, and *RC7G0563900 (ANS)* may be related to the deepening of ‘Sunset Babylon Eyes’ blotch in the later stages, but the relative expression level of these four enzyme genes was not much different from those of the non-blotch parts. Two enzyme genes, *RC0G0031100 (FLS)* and *RC5G0593900 (A3GT)*, significantly highly expressed in the non-blotch parts at S2 and S3, respectively; hence they may be involved in the synthesis of flavonol glycosides in the non-blotch parts. Three enzyme genes, *RC7G0058400 (F3’H)*, *RC6G0470600 (DFR)* and *RC7G0212200 (ANS)*, all significantly highly expressed in the blotch parts at S2 and S4, which were supposed to be the key enzyme genes for the early pigmentation and color deepening of blotch in the later stages (Fig. 5).

*RC2G0093100 (MYB)* significantly highly expressed in the blotch part at S2, but also highly expressed in the non-blotch part at S3. *RC2G0406300 (AP2)* significantly highly expressed in the blotch part at S2, however, at S4, its relative expression level of the blotch part was lower than that of the non-blotch part. These two regulatory genes may be involved in the regulation of the early pigmentation in blotch parts, or also be related to the regulation of the late pigmentation in non-blotch parts at the same time. The relative expression levels of six regulatory genes, including *RC7G0019000 (MYB)*, *RC1G0363600 (WRKY)*, *RC5G0166100 (WRKY)*, *RC0G0179800 (NAC)*, *RC1G0093800 (NAC)*, and *RC0G0030000 (DOF)*, were always higher of the blotch parts than that of the non-blotch parts from S2 to S4. It is noteworthy that while *RC7G0058400 (F3’H)*, *RC6G0470600 (DFR)* and *RC7G0212200 (ANS)* significantly highly expressed at S2 and S4 in the blotch parts, there were corresponding trends of the relative expression levels of *RC7G0019000 (MYB)* and *RC1G0363600 (WRKY)*. Hence, *RC7G0019000 (MYB)* and *RC1G0363600 (WRKY)* may be the key regulatory genes for the early pigmentation and color deepening of blotch in the later stages. In contrast, the relative expression level of the rest two genes, *RC5G0279600 (TCP)* and *RC2G0471400 (WD40)*, in blotch parts were lower than those in non-blotch parts from S2 to S4. In that case, these two genes may negatively regulate the pigmentation of blotch (Fig. 5).

### Promoter sequences analysis of genes most likely to influence the blotch pigmentation

The promoter sequences, which were located 2000 nt upstream of ATG, of three enzyme genes and one regulatory gene were obtained from the reference genome (https://www.ncbi.nlm.nih.gov/assembly/GCF_002994745.2). All three enzyme genes’ promoters had the cis-acting regulatory element of MYB recognition site (MRS), MYB binding site (MBS) or MYB recognition element (MRE) (Supplementary Fig. 6a, b and c). This meant that *RC7G0058400 (F3’H)*, *RC6G0470600 (DFR)* and *RC7G0212200 (ANS)* were all likely to be regulated by MYB transcription factor. Meanwhile, the promoter sequences of *RC7G0058400 (F3’H)*, *RC6G0470600 (DFR)* and *RC7G0019000 (MYB)* all had the cis-acting regulatory element of W box or WRKY recognition element (WRE), so all of them might be regulated by the transcription factor of WRKY (Supplementary Fig. 6a, b and d).

## Discussion

### A potential model plant for blotch formation study

To elucidate the differences of composition and content of anthocyanins between blotch and non-blotch parts, most researches on blotch pigmentation have chosen the plant materials which have color blotch and uncolor (i.e., white) non-blotch parts, or exactly the opposite [6, 8, 9, 50–59]. However, it is difficult to know about the profile of other flavonoids and other pigments such as carotenoids (Supplementary Table S4). Even if the materials were used with different colors in both blotch and non-blotch parts, there were still few reports comparing anthocyanins, flavones and flavonols, as well as carotenoids at the same time between these two parts [60–63] (Supplementary Table S4). In this study, the flavonoids and carotenoids were identified and characterized together in a *Rosa* Hulthemia hybrid ‘Sunset Babylon Eyes’ with rose-red to dark red blotch and yellow non-blotch parts. According to the identification and characterization, this cultivar could be regarded as a potential model plant for blotch formation research for the following three reasons.

Firstly, the anthocyanin composition of its petal was quite simple. Only one anthocyanin: cyanidin 3,5-*O*-diglucoside (Cy3G5G) was found in the petal’s blotch. As for *Paeonia suffruticosa* [50], *Viola* × *wittrockiana* Gams [6]. and *Clarkia gracilis* [5], it was hard to clarify the anthocyanin biosynthesis mechanism between blotch and non-blotch parts because either the anthocyanins were detected in both of two parts, or there was more than one anthocyanin component existing in the blotch (Supplementary Table S4). Secondly, the petal’s flavonoid components were also quite simple. No flavone but only flavonols (quercetin and kaempferol glycosides) were detected in both of the blotch and non-blotch parts. And this result was consistent with the previous finding in the genus *Rosa* [42]. Compared with *Paeonia suffruticosa* cultivar ‘High Noon’ which had both of flavones and flavonols in petals [60] (Supplementary Table S4), *Rosa* ‘Sunset Babylon Eyes’ could directly start from the research on flavonol biosynthesis pathway. Thirdly, a variety of different carotenoids played an assisted role in the coloration of petals by adding orange or yellow hue as in other plants’ flowers [29, 64, 65]. Notably, we identified four more carotenoids in ‘Sunset Babylon Eyes’ than in genus *Mimulus* [63], it could help explain much more complex blotch pigmentation mechanism in plants. Collectively, the surprising simpleness of flavonoids and diversity of carotenoids in petals of ‘Sunset Babylon Eyes’ made it can be used to study the mechanism of petal blotch pigmentation as a potential model plant.

### Molecular mechanism comparisons of blotch formation

*F3’H* is the key enzyme gene for the synthesis of cyanidin pigments by generating eriodicyol from naringenin or generating dihydroquercetin from dihydrokaempferol. Also, both of *DFR* and *ANS* are critical genes for anthocyanin biosynthesis. Despite of the differences, all these three enzyme genes played a vertical role in the molecular mechanism of different blotch color formation in different species [6, 9, 50, 51, 60, 61, 63] (Supplementary Table S4). In ‘Sunset Babylon Eyes’, *RC7G0055400 (F3’H)*, *RC6G0467567 (DFR)*, and *RC7G0212200 (ANS)* which all expressed significantly highly may be the most significant enzyme genes that directly facilitate the pigmentation of blotch.

MYB belongs to a common transcription factor family that regulates anthocyanin synthesis. *MYB*, especially *R2R3-MYB* participated in almost all the blotch formation mechanism in different species by activating, enhancing or inhibiting [8, 50, 53–56, 58, 60–62, 66–68] (Supplementary Table S4). WRKY is also an important transcription factor that found to be involved in regulating anthocyanin biosynthesis. Overexpression of *MdWRKY11* could promote the expression of *F3H*, *FLS*, *DFR*, *ANS* and *UFGT*, thereby increasing the accumulation of flavonoids and anthocyanin in apple calli [69]. The interaction of *PyWRKY26* and *PybHLH3* could co-target the promoter of *PyMYB114*, which activated the expression of *PyDFR*, *PyANS*, and *PyUFGT*, and therefore resulted in anthocyanin accumulation in red-skinned pear [70]. *StWRKY13* could not only enhance the role of *StAN2* (belongs to MYB family) in promoting anthocyanin biosynthesis in tobacco, but also interact with the promoters of *StCHS*, *StF3H*, *StDFR* and *StANS* genes, thereby enhancing their activation of anthocyanin formation in coloured potato tubers [71]. Until now, no research on WRKY influences blotch formation has been reported.

In ‘Sunset Babylon Eyes’, *MYB (RC7G0019000)* and *WRKY (RC1G0360567)* significantly highly expressed in blotch parts at S2 and S4, and their relative expression level trends matched that of *RC7G0055400 (F3’H)*, *RC6G0467567 (DFR)* and *RC7G0212200 (ANS)*. The cis-acting regulatory elements analysis of promoter sequences indicated that MYB (RC7G0019000) and WRKY (RC1G0360567) may bind to the promoters of critical enzyme genes or WRKY (RC1G0360567) may bind to the promoter of *MYB (RC7G0019000)* to activate the anthocyanin accumulation in rose blotch.

### The roles of developmental, environmental and hormonal factors played in blotch formation

Anthocyanins take on a critical role for the attraction of pollinators and enhancing tolerance to abiotic stress (e.g., drought and cold) by absorbing excess light in angiosperms [72, 73]. The broad functionality of anthocyanins requires sophisticated regulation of the anthocyanin biosynthesis pathway to allow proper localization, timing, and optimal intensity of pigment deposition [72]. To find clues to the upstream regulatory genes of blotch formation in rose, we analyzed the genes associated with developmental program, environmental cues, or plant hormones. Of 1109 genes, 22 genes were found with corresponding expression to *WRKY (RC1G0363600)* or *MYB (RC7G0019000)*.12 genes may be positive regulatory genes of *WRKY (RC1G0363600)* or *MYB (RC7G0019000)* while 10 genes may be negative regulators (Supplementary Fig. 7, Supplementary Table S5).

Genes involved in developmental program, environmental cues, or plant hormones have a positive or negative effect on anthocyanin accumulation in different species [9, 74]. Silencing *PaMADS2*, *PaMADS4* and *PaMADS7* inhibited fruit ripening and decreased the anthocyanin content in sweet cherry [74]. MADS-box genes (*ScAG* and *ScAGL11*) negatively regulated anthocyanin biosynthesis in cineraria ray florets, and their differential expression influenced the bicolour pattern appearance [9]. One GATA might act as a positive regulator of anthocyanin accumulation in petals of *Camellia japonica* whereas GATA became a negative regulator of anthocyanin synthesis during *Lycoris radiata* petal development stages [75, 76]. Genes from the same family may have the opposite trends of expression in the same plant [77]. Stress-related protein HSF, which might regulate the ripening process by the regulation of ethylene and other hormones and anthocyanin biosynthesis, showed differential expression (up or down) in the post-ripening samples in apples [77]. Exogenous application of hormones, calcium and environmental changes can also effect anthocyanin accumulation [78–81]. Exogenous application of calcium could promote apple coloring [78]. Far-red addition resulted in a decrease in the levels of carotenoids and anthocyanins [79, 80]. A variety of genes act as anthocyanin repressors in different plants and much regulation of anthocyanin production involves signal-induced degradation or sequestration of the repressors from the MBW protein complex [72, 81]. In *Hibiscus syriacus*, IAA (auxin-responsive protein IAA) could bind with ARFs to remove their repressive action on anthocyanin biosynthesis genes, while JAZ degradation caused the transcriptional activation of MBW complex and thereby resulting in color flower [81]. In addition to repressors, regulatory genes can act as activators to promotes anthocyanin accumulation [82–85]. The bZIP transcription factor *MdHY5* promoted anthocyanin accumulation by regulating expression of the *MdMYB10* and downstream anthocyanin biosynthesis genes in apple [82]. The C2H2-type zinc finger gene *JAGGED (JAG)* could regulate cell differentiation and flower morphological development in *Arabidopsis thaliana*. Overexpression of *MdZAT5* (a C2H2-type zinc finger gene) promoted anthocyanin accumulation in apple calli and *Arabidopsis thaliana* [83, 84]. *LhGST* was positively correlated with anthocyanin accumulation in lilies [85]. Consequently, the relationship between developmental program, environmental cues, or plant hormones and anthocyanin accumulation is complex and there are still many unanswered questions. However, we can still find some clues to the positive or negative effects of developmental program, environmental cues, or plant hormones on rose blotch formation (Supplementary Fig. 7, Supplementary Table S5).

## Conclusions

**Fig. 6:**
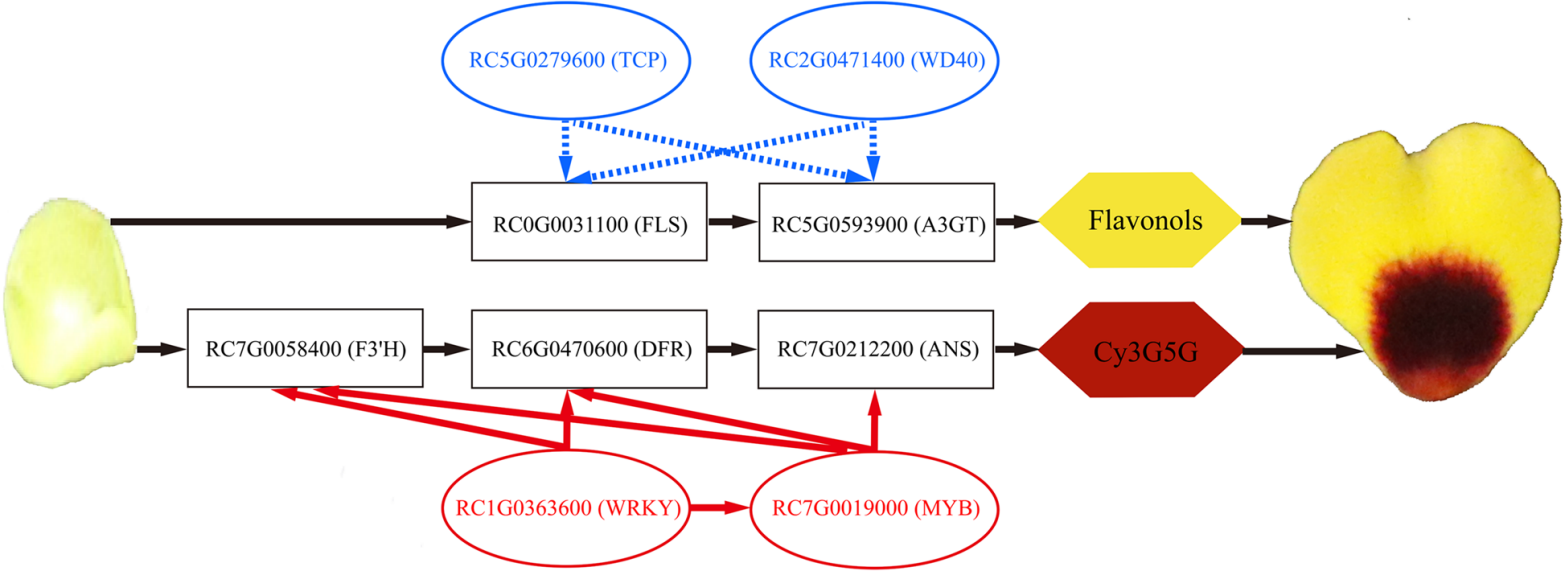
Model depicting the pigmentation of petal blotch in rose

Metabolome and transcriptome data were used to reveal the formation of blotch in rose petals. Variation in accumulation of the only detected anthocyanin metabolite (cyanidin 3,5-*O*-diglucoside) in different regions of petals contributed to the blotch formation. In addition, 11 flavonol glycosides were responsible for the yellow background color while 12 carotenoids brighten the petal color. In an analysis of the anthocyanin biosynthesis pathway, 9 structural genes (in particular, *F3’H, DFR* and *ANS*) and 10 TFs (especially *MYB, WRKY, TCP* and *WD40*) were identified. Analysis of cis-acting regulatory elements of promoter sequences suggested that MYB (RC7G0019000) and WRKY (RC1G0363600) may bind to the promoters of *RC7G0058400 (F3’H)*, *RC6G0470600 (DFR)* or *RC7G0212200 (ANS)*. Also WRKY (RC1G0363600) may bind to the promoter of *MYB (RC7G0019000)* to activate anthocyanin accumulation in rose blotch (Fig. 6). These findings can help elucidate the molecular mechanism and regulatory networks of anthocyanin biosynthesis in rose and provide a biological basis for breeding novel rose cultivars.

## Methods

### Sample collection

The rose cultivar ‘Sunset Babylon Eyes’ grew under normal field conditions, including irrigation, fertilization, and disease and pest control in Beijing Academy of Forestry and Landscape Architecture, Beijing, China. Rose flower development stages were classified as previously described with slight modifications [8, 47, 48]. Flowers were collected at five developmental stages: colorless bud petal stage at which the petal does not have blotch (S1); initially colored bud petal stage at which the diameter of bud is 8.0 ± 0.5 mm, and the length and width of blotch are 5.0 ± 0.5 mm and 4.0 ± 0.5 mm respectively (S2); colored bud petal stage at which the diameter of bud is 12.0 ± 0.5 mm, and the length and width of blotch are 7.5 ± 0.5 mm and 6.0 ± 0.5 mm respectively (S3); initiating blooming stage (S4); and blooming stage (S5).

After accurately measured for color, the collected dried petals were used for pigment analysis, while other fresh samples were stored at − 80 °C for subsequent transcriptome sequencing and quantitative real-time PCR analysis.

### Determination of colors

The colors of the petal samples were detected with a spectrophotometer NF555 (Nippon Denshoku Industries Co., Ltd., Tokyo, Japan) under the condition that the indoor light was sufficient but not dazzling, and the light source could be kept stable for a long time. For each sample, three petals were detected and each petal was measured five times. The values of L^*^, a^*^ and b^*^ were collected and processed by ColorMate software [42].

### Extraction and detection of flavonoids

The extraction and flavonoid analysis were carried out as described by a previous study [42], with minor modification. Briefly, about 20 mg of freeze-dried petal powder was extracted with 4 mL of extracting solution (50: 49.5: 0.5, v/v/v, acetonitrile: water: formic acid) using ultrasonication and centrifugeing. The HPLC analysis was performed on Agilent 1260 Infinity II HPLC-DAD (Agilent Technologies, Palo Alto, CA, USA). The liquid chromatograph was equipped with a Cosmosil Cholester column (4.6 mm × 250 mm, 5 μm) (Nacalai Tesqye, Inc., Nakagyo-ku, Kyoto, Japan). The flow rate, injected volume and column temperature was 0.6 mL/min, 10 μL and 30 °C, respectively. The chromatograms were extracted at 350 nm for flavonols and 520 nm for anthocyanins. Flavonols and anthocyanins were quantitatively analyzed by external standard method. Calibration equations for cyanidin 3,5-*O*-diglucoside and quercetin 3-*O*-galactoside were y = 22,887x-96.484 (*R*^2^ = 0.9987) and y = 46,796x + 10.026 (*R*^2^ = 0.9996), respectively. The following analysis of mass spectrometry was performed on Waters I-Class UPLC/Xevo TQ MS (Waters Corporation, Milford, MA, USA). Identification of flavonols and anthocyanins were based on standards and a previous study [42].

### Extraction and detection of carotenoids

The extraction and HPLC analysis of carotenoids were performed as described by a previous study [42], with minor modification. Briefly, 200 mg freeze-dried petal powder was extracted with 2 mL methanol and then 2 mL *n*-hexane and 1 mL NaCl solution (10%, w/v) were added. Next, the upper organic phase was collected and the bottom layer was re-extracted with 1 mL of *n*-hexane: diethyl ether (volume ratio = 3:1) until the sample turned colorless. The combined extracts were evaporated to dryness in a nitrogen blower and saponified with 1 mL of 6% KOH methanolsolution. Then the mixture was extracted with 1 mL of NaCl solution (10%, w/v) and 1 mL of *n*-hexane: ether (3:1, volume ratio) until the sample appeared colorless. The extracted supernatants were concentrated to dryness with Concentrator plus vacuum centrifugal concentrator (Eppendorf Group, Hamburg, Germany). The saponified carotenoid dry powder was reconstituted with methanol-methyl tert-butyl Base ether (1:1, v/v) prior to HPLC analysis. To avoid carotenoid degradation, 0.1% BHT (w/v) was added to the solvent extracts. HPLC analysis was performed on Agilent 1260 Infinity II HPLC-DAD (Agilent Technologies, Palo Alto, CA, USA). The flow rate, injection volume and column oven temperature was 1 mL/min, 100 *u*L and 25 °C, respectively. The detection wavelength of carotenoids was 450 nm. Carotenoids were quantitatively analyzed by external standard method. Calibration equations for 13/13’*Z*-Neoxanthin was y = 288,930x-614.38 (*R*^2^ = 0.9999).

The following analysis of mass spectrometry was performed on Waters I-Class UPLC/Xevo TQ MS (Waters Corporation, Milford, MA, USA). Identification of carotenoids was based on standards and a previous study [42].

### Transcriptome sequencing

The total RNA was extracted with the Invitrogen TRIzol (Thermo Fisher Scientific, Waltham, MA, USA). The quality and integrity of RNA were assessed with a 1% agarose gel, NanoDrop 2000 (Thermo Fisher Scientific, Waltham, MA, USA) and Agilent 2100 Bioanalyzer (Agilent Technologies, Palo Alto, CA, USA). The construction of the libraries and RNA-Seq were carried out on an Illumina Novaseq™ 6000 platform (Illumina, Inc., San Diego, CA, USA) by HT Health Biotechnology Corporation (Beijing, China).

After removing low-quality reads, undetermined reads and reads containing prime/ adaptor, raw data were filtered as clean data for the following analyses. Clean reads were mapped to the genome of *R. chinesis* (https://pubmed.ncbi.nlm.nih.gov/29713014/) by HISAT2 software (version: hisat2–2.0.4) and then the mapped reads were assembled using StringTie (version: stringtie-1.3.4d.Linux_x86_64) with default parameters. Subsequently, all transcriptomes were merged to reconstruct a comprehensive transcriptome using gffcompare software (version: gffcompare-0.9.8.Linux_x86_64). The expression of mRNA was calculated by FPKM (Fragments Per kb Per Million Reads) method. The differentially expressed genes (DEG) were selected with thresholds of fold change > 2 or fold change < 0.5, *p* value< 0.05 by DESeq2 tool, and then used for the GO and KEGG [86] analyses.

### Quantitative real-time PCR (qRT-PCR) analysis

Gene expression analysis was conducted by qRT-PCR as described by Gu et al. [8]. The primers, including reference gene tubulin, used for qRT-PCR analysis are listed in Supplementary document (Supplementary Table S6). Total RNA was prepared according to the chapter ‘Transcriptome sequencing’ and cDNA synthesis was conducted using the Thermo Scientific Maxima Reverse Transcriptase (Thermo Fisher Scientific, Waltham, MA, USA). Quantitative assays were carried out using the Roche LightCycler480 II RealTime PCR system (Roche Molecular Systems, Inc., Rotkreuz, Switzerland) and the ABI StepOnePlus™ RealTime PCR system (Thermo Fisher Scientific,Waltham, MA, USA) as described by the manufacturers. Three biological replicates with triplicate technical replicates were analyzed for each sample.

### The key cis-acting regulatory elements of promoter sequences analysis

Promoter sequences of the obtained key genes were analyzed using Plant-CARE online software (http://bioinformatics.psb.ugent.be/webtools/plantcare/html/) to predict the possible cis-acting regulatory elements.

### Statistical analysis

Data were analyzed using IBM SPSS Statistics 25.0 (IBM, Armonk, NY, USA), MassLynx (Waters Corporation, Milford, MA, USA) and Origin 9.0 (OriginLab Corporation, Northampton, MA, USA). All assays were performed in triplicate. Relative expression level of candidate genes by qRT-PCR were compared by analysis of variance (ANOVA).

## Supplementary Information

**Additional file 1: Supplementary Fig. S1.** HPLC chromatograms of flavonoid detected at 525 nm and 350 nm. **Supplementary Fig. S2.** Difference analysis of gene expression between blotch and non-blotch parts. **Supplementary Fig. S3.** Veen analysis of gene expression between blotch and non-blotch parts. **Supplementary Fig. S4.** GO classification of differentially expressed genes. **Supplementary Fig. S5.** KEGG enrichment of differentially expressed genes. **Supplementary Fig. S6.** Promoter sequences and key cis-acting regulatory elements of genes most likely to influence the blotch pigmentation in rose. **Supplementary Fig. S7.** DEGs associated with developmental program, environmental cues, or plant hormones. **Supplementary Table S1.** Contents of pigments. **Supplementary Table S2.** Identification of carotenoids. **Supplementary Table S3.** RNA sequencing data and corresponding quality control. **Supplementary Table S4.** Different blotch formation in different species. **Supplementary Table S5.** Other DEGs identified in anthocyanin biosynthesis-related pathways. **Supplementary Table S6.** Primers required for candidate unigenes qRT-PCR expression.

## Data Availability

All data generated or analyzed during this study are included in this published article and its Supplementary information file. The raw RNA-seq data have been submitted to the SRA database under accession number PRJNA870267, and they can also be freely available at: https://www.ncbi.nlm.nih.gov/sra/PRJNA870267.

## Abbreviations

MYB: v-myb avian myeloblastosis viral oncogene homolog
R2R3-MYB: MYB with 2 serial and nonredundant imperfect sequence repeats
bHLH: basic helix–loop–helix
WD40: WD repeat
WRKY: transcription factors contain a WRKYGQK heptapeptide at the N-terminal end and a zinc-finger motif (CX4-5CX22-23HXH or CX7CX23HXC) at the C-terminal end
NAC: NAM, ATAF1/2, and CUC2
DOF: DNA binding with one finger
AP2: APETALA2/ethylene-responsive element binding proteins
TCP: TEOSINTE BRANCHED1, CYCLOIDEA, and PROLIFERATING CELL FACTORS
HPLC-DAD: high-performance liquid chromatography-diode array detection
UPLC-TQ-MS: ultra-high performance liquid chromatography coupled with triple quadrupole mass
KEGG: Kyoto Encyclopedia of Gene and Genome
GO: Gene Ontology
