# Supplementary material for: Metabolic profile and transcriptome reveal the mystery of petal blotch formation in rose
Source: BMC Plant Biol. 2023 Jan 20;23:46. doi: 10.1186/s12870-023-04057-6 (PMC9854060; doi:10.1186/s12870-023-04057-6)
Supplement: Supplementary file 1 — Additional file 1: Supplementary Fig. S1. HPLC chromatograms of flavonoid detected at 525 nm and 350 nm. Supplementary Fig. S2. Difference analysis of gene expression between blotch and non-blotch parts. Supplementary Fig. S3. Veen analysis of gene expression between blotch and non-blotch parts. Supplementary Fig. S4. GO classification of differentially expressed genes. Supplementary Fig. S5. KEGG enrichment of differentially expressed genes. Supplementary Fig. S6. Promoter sequences and key cis-acting regulatory elements of genes most likely to influence the blotch pigmentation in rose. Supplementary Fig. S7. DEGs associated with developmental program, environmental cues, or plant hormones. Supplementary Table S1. Contents of pigments. Supplementary Table S2. Identification of carotenoids. Supplementary Table S3. RNA sequencing data and corresponding quality control. Supplementary Table S4. Different blotch formation in different species. Supplementary Table S5. Other DEGs identified in anthocyanin biosynthesis-related pathways. Supplementary Table S6. Primers required for candidate unigenes qRT-PCR expression. [file 12870_2023_4057_MOESM1_ESM.pdf]

Supplementary information

Supplementary Figure S1. HPLC chromatograms of flavonoid detected at 525 nm and 350 nm.

Supplementary Figure S2. Difference analysis of gene expression between blotch and non-blotch parts.

Supplementary Figure S3. Veen analysis of gene expression between blotch and non-blotch parts.

Supplementary Figure S4. GO classification of differentially expressed genes.

Supplementary Figure S5. KEGG enrichment of differentially expressed genes.

Supplementary Figure S6. Promoter sequences and key cis-acting regulatory elements of genes most likely to influence the blotch pigmentation in rose.

Supplementary Figure S7. DEGs associated with developmental program, environmental cues, or plant hormones。

Supplementary Table S1. Contents of pigments.

Supplementary Table S2. Identification of carotenoids.

Supplementary Table S3. RNA sequencing data and corresponding quality control.

Supplementary Table S4. Different blotch formation in different species.

Supplementary Table S5. Other DEGs identified in anthocyanin biosynthesis-related pathways.

Supplementary Table S6. Primers required for candidate unigenes qRT-PCR expression.

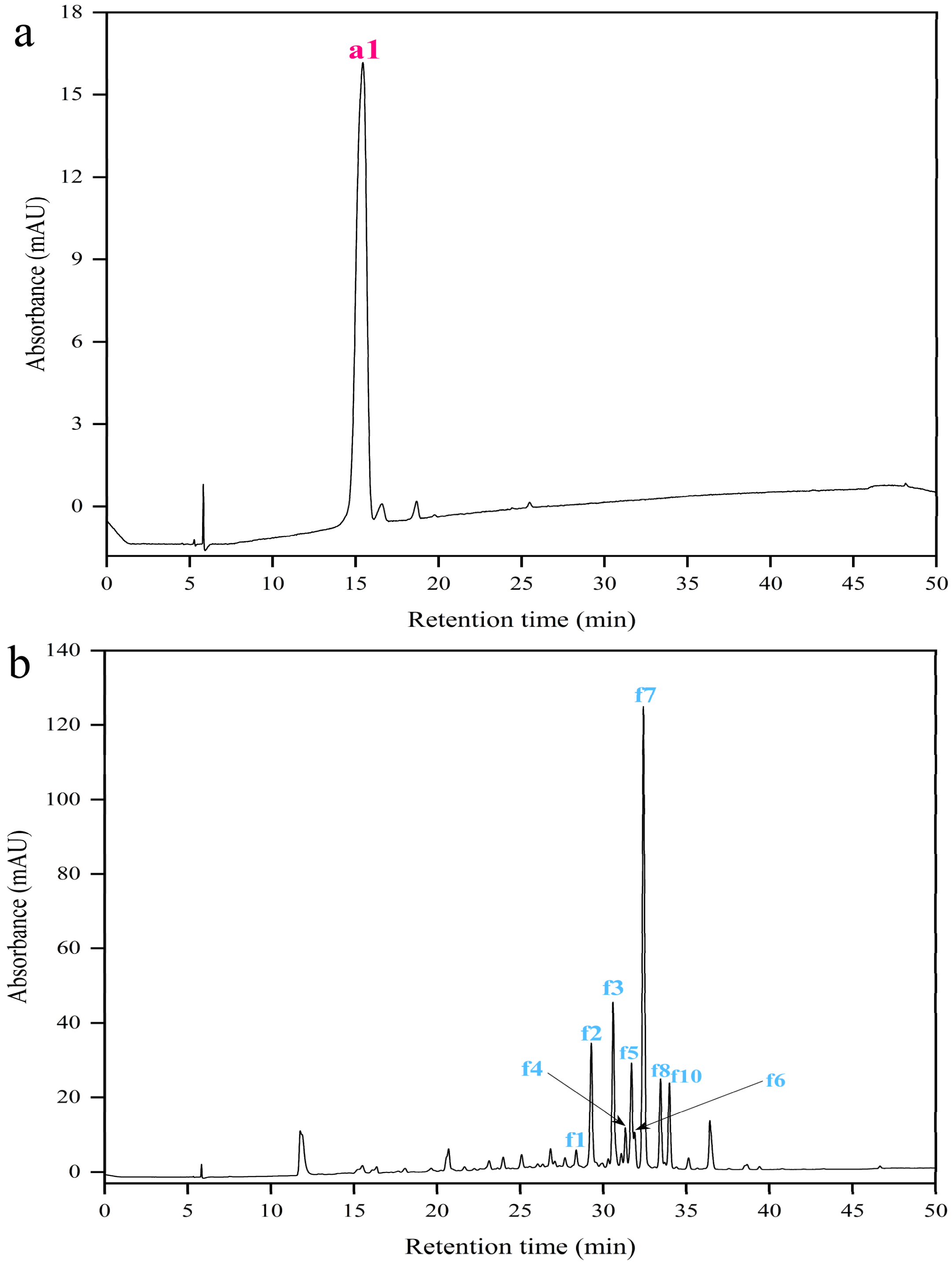

Supplementary Figure S1. HPLC chromatograms of flavonoid detected at 525 nm (a) and 350 nm (b).

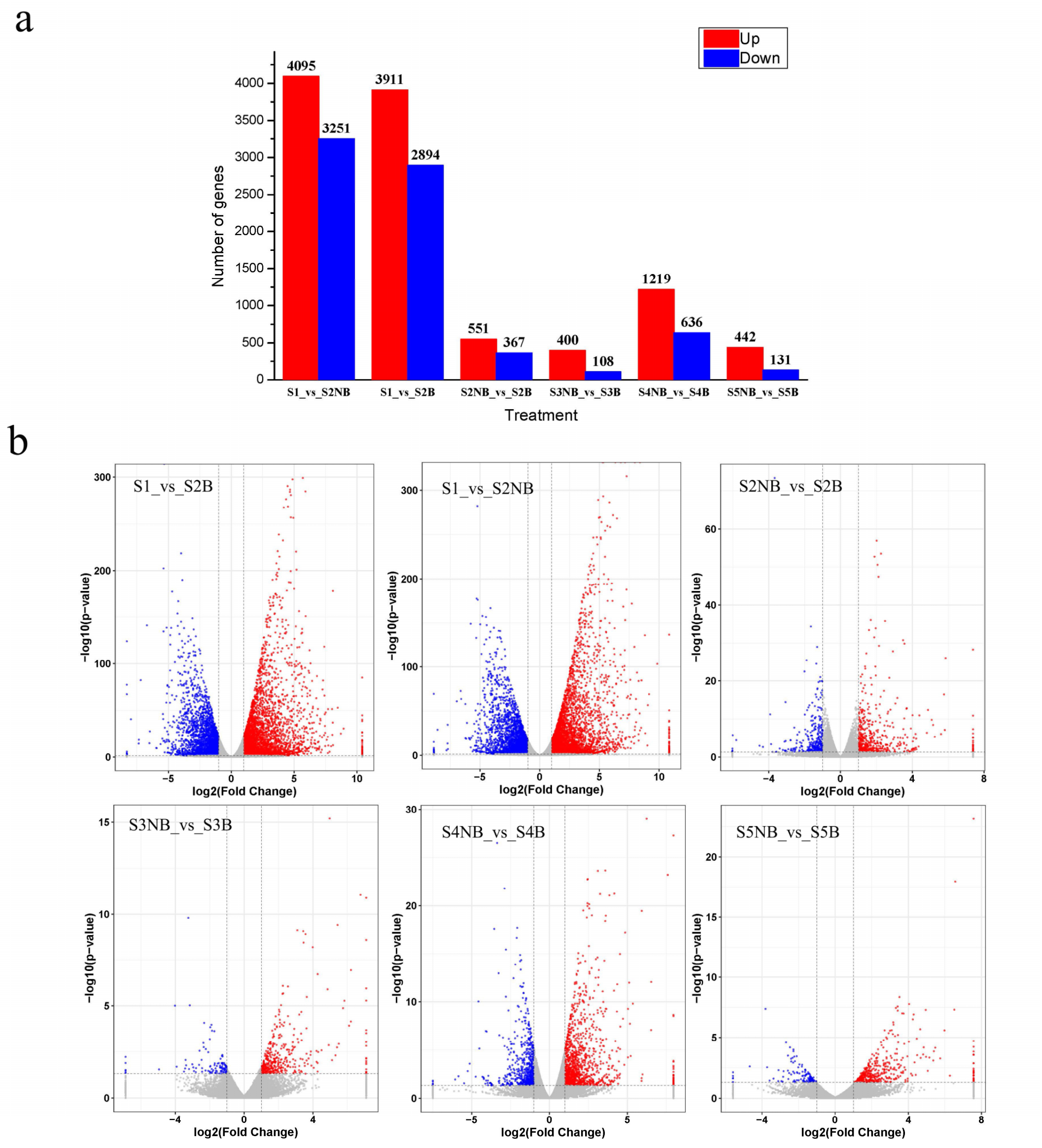

Supplementary Figure S2. Difference analysis of gene expression between blotch and non-blotch parts. (a) Comparison of the number of up- and down- regulated genes. (b) Volcano plots between treatments and control. Red and blue points represent up- and down- regulated genes, respectively. Block points represent no difference genes.

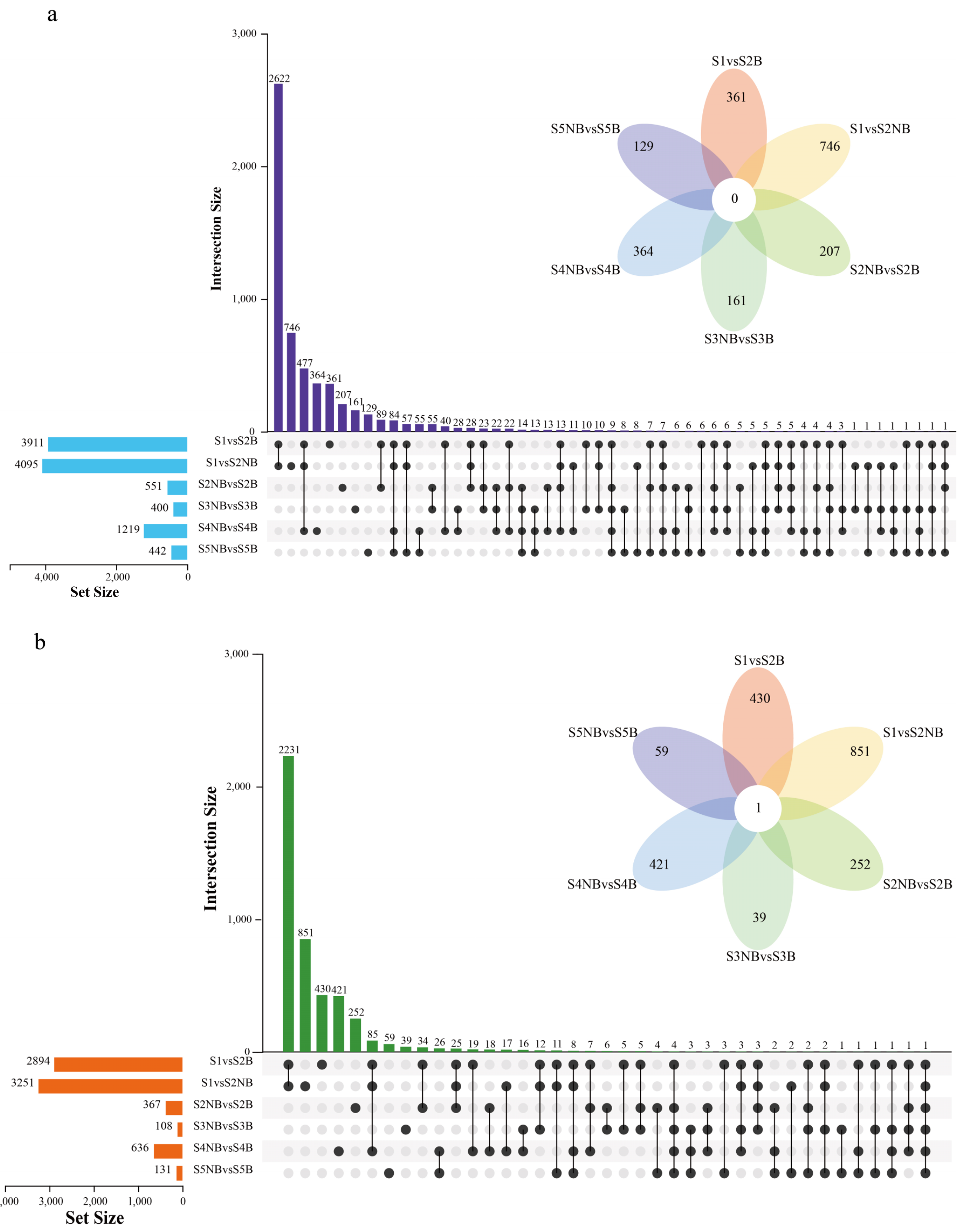

Supplementary Figure S3. Veen analysis of gene expression between blotch and non-blotch parts. (a) Veen diagram analysis of upregulated DEGs from S1 to S5 stages. (b) Veen diagram analysis of downregulated DEGs from S1 to S5 stages.

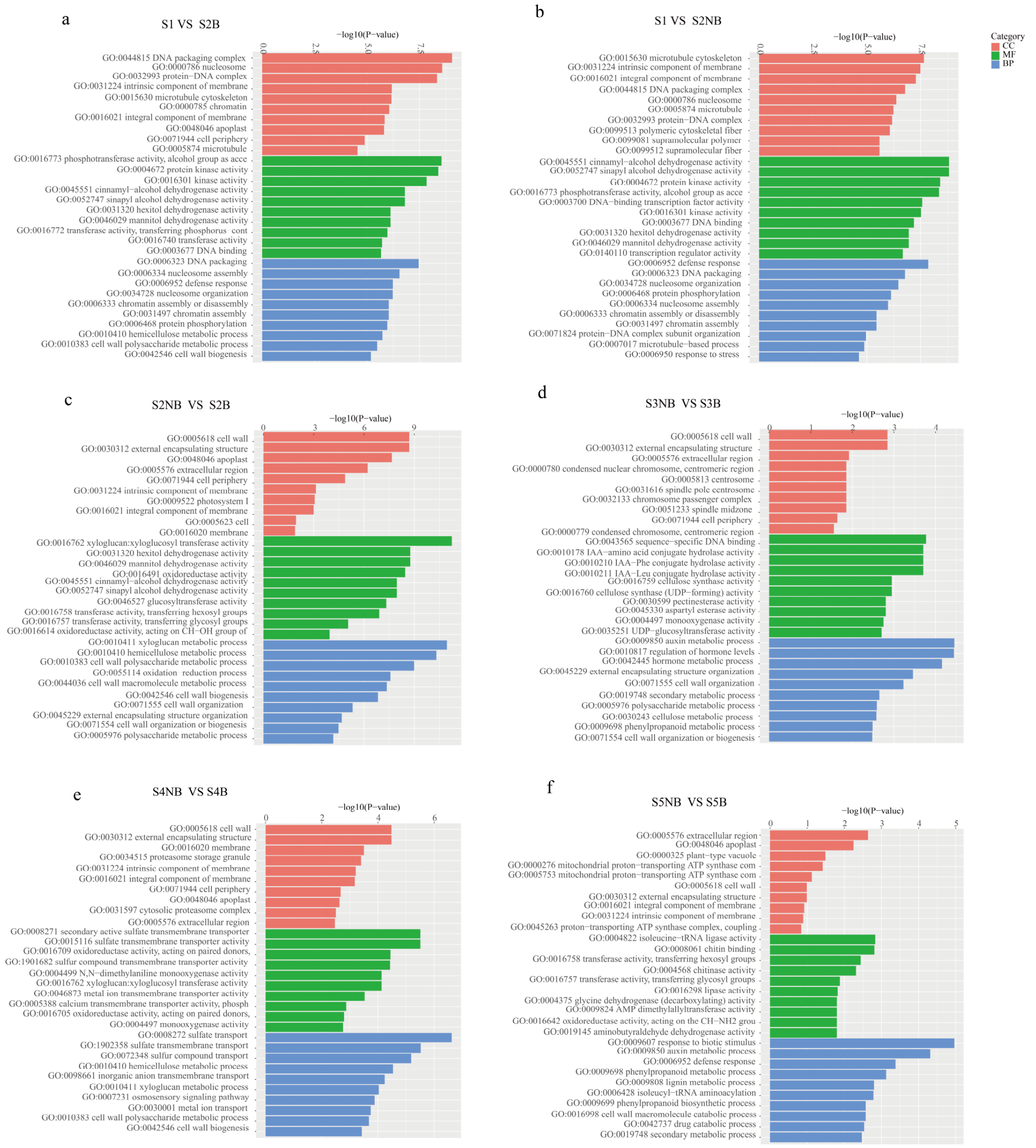

Supplementary Figure S4. GO classification of differentially expressed genes. (a) S1_vs_S2B. (b) S1_vs_S2NB. (c) S2NB_vs_S2B. (d) S3NB_vs_S3B. (e) S4NB_vs_S4B. (f) S5NB_vs_S5B.

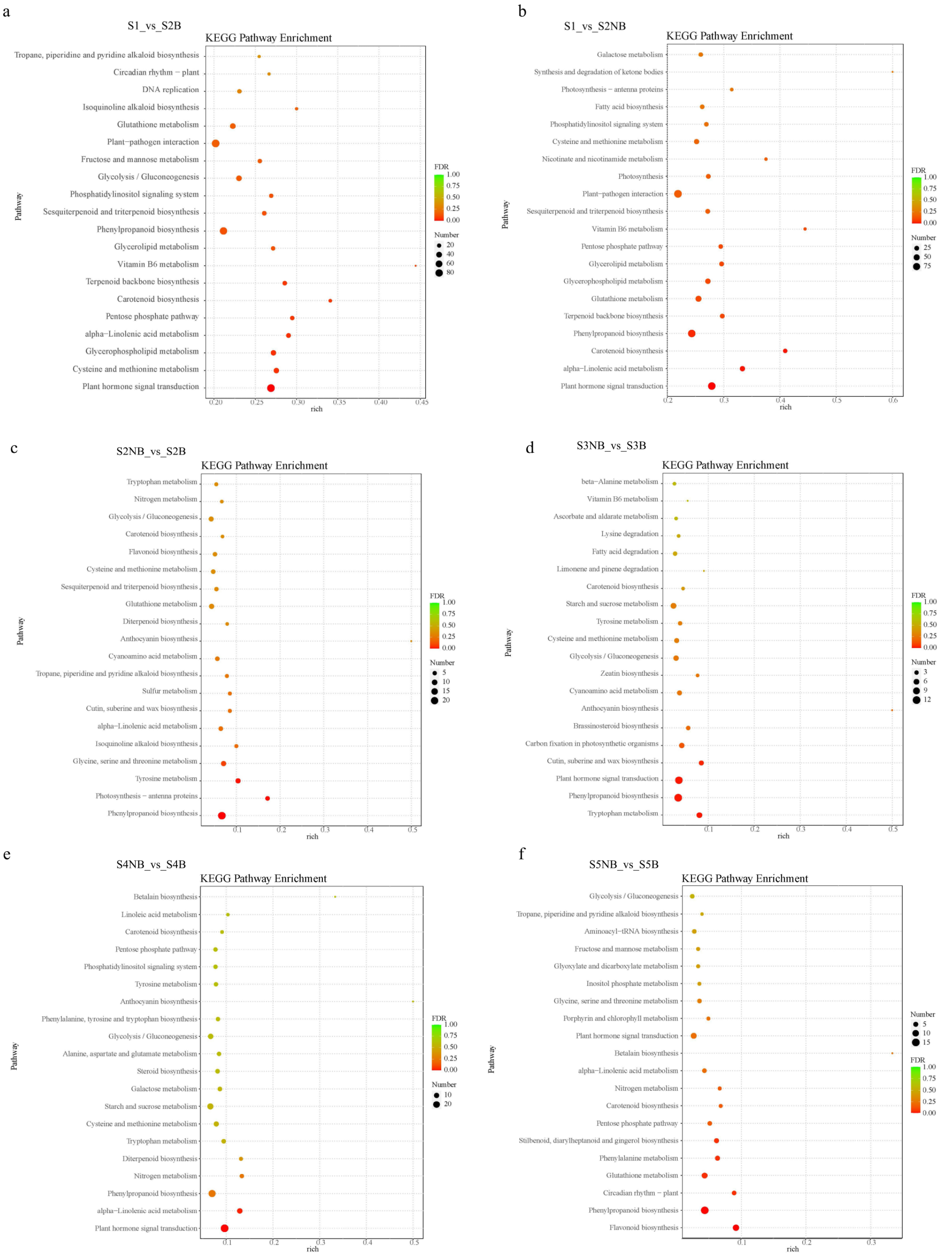

Supplementary Figure S5. KEGG enrichment of differentially expressed genes. (a) S1_vs_S2B. (b) S1_vs_S2NB. (c) S2NB_vs_S2B. (d) S3NB_vs_S3B. (e) S4NB_vs_S4B. (f) S5NB_vs_S5B.

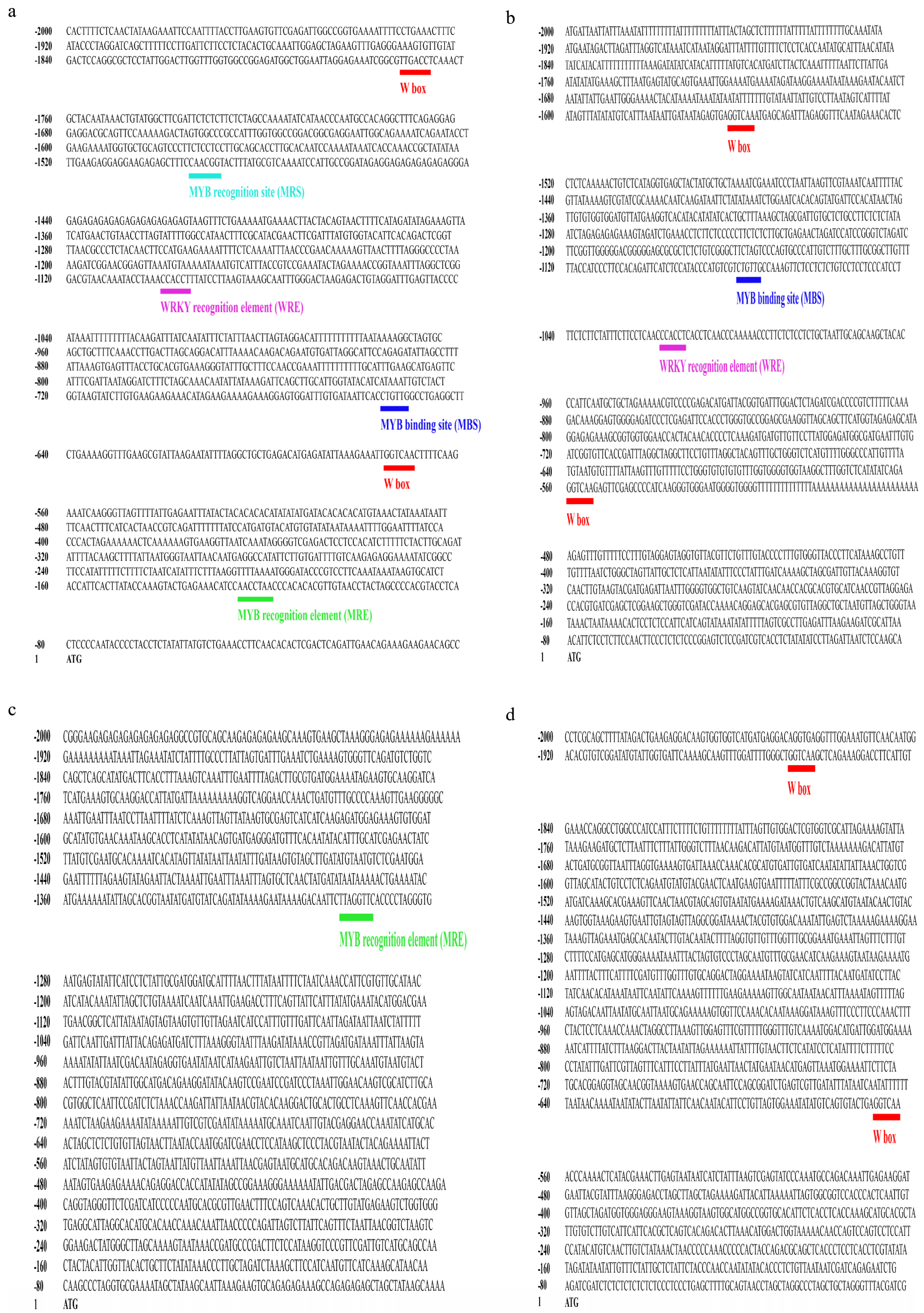

Supplementary Figure S6. Promoter sequences and key cis-acting regulatory elements of genes most likely to influence the blotch pigmentation in rose. (a) Promoter sequence and key cis-acting regulatory elements of *RC7G0058400 (F3'H)*. (b) Promoter sequence and key cis-acting regulatory elements of *RC6G0470600 (DFR)*. (c) Promoter sequence and key cis-acting regulatory element of *RC7G0212200 (ANS)*. (d) Promoter sequence and key cis-acting regulatory elements of *RC7G0019000 (MYB)*.

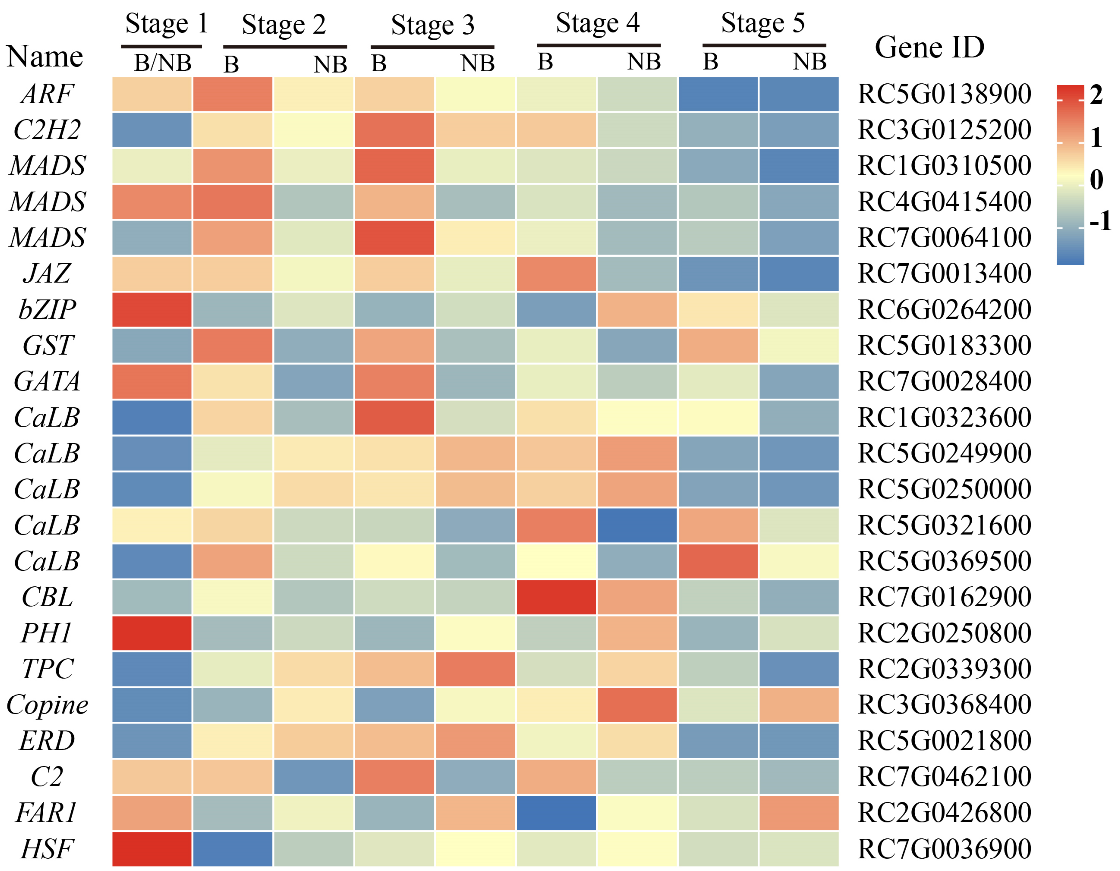

Supplementary Figure S7. DEGs associated with developmental program, environmental cues, or plant hormones. B: the blotch part of the petal. NB: the non-blotch part of the petal.

Supplementary Table S1 Contents of pigments

Stages / Contents (mg/g DW) / Pigments	S1	S2B	S2NB	S3B	S3NB	S4B	S4NB	S5B	S5NB
a1	0.0000±0.0000	1.0773±0.0087	0.0000±0.0000	1.8143±0.0583	0.0000±0.0000	7.9706±0.2639	0.0000±0.0000	7.1104±0.0870	0.0000±0.0000
f1	0.0734±0.0205	0.1800±0.0023	0.0947±0.0153	0.1892±0.0150	0.1179±0.0158	0.1115±0.0047	0.1539±0.0158	0.0991±0.00001	0.1386±0.0222
f2	1.1899±0.0165	1.2443±0.0133	2.1071±0.2370	1.4226±0.1088	2.6463±0.2196	0.0947±0.0014	1.3384±0.0358	0.4671±0.0002	1.0107±0.0196
f3	1.6552±0.1041	1.6012±0.0063	2.4592±0.2640	1.7874±0.1100	0.1328±0.0142	0.0361±0.0006	0.1865±0.0069	0.0519±0.0012	0.1715±0.0059
f4	0.2427±0.0142	0.2418±0.0021	0.1573±0.0214	0.3553±0.0275	3.3743±0.2870	0.0549±0.0004	1.8191±0.0641	0.8729±0.0048	1.5679±0.0404
f5	0.2967±0.0468	0.7020±0.0100	0.2622±0.0315	1.1467±0.0732	0.5721±0.0590	0.9438±0.0075	1.2915±0.0424	0.6143±0.0067	1.2162±0.0323
f6	0.1887±0.0076	0.2546±0.0006	0.4184±0.0495	0.2954±0.0224	0.6889±0.0784	0.6603±0.0063	2.0723±0.0708	1.9253±0.0076	1.8255±0.1356
f7	2.5669±0.0167	3.9861±0.0053	4.5394±0.4797	5.1182±0.2933	0.4738±0.0488	2.1265±0.0197	0.0493±0.0855	0.0000±0.0000	0.0000±0.0000
f8	0.2964±0.0082	0.6971±0.0023	0.7429±0.0833	0.9117±0.0551	7.2766±0.6312	3.1468±0.0157	5.5729±0.1812	2.7233±0.0200	4.5164±0.1039
f9	1.0041±0.1899	0.3166±0.0037	0.7952±0.0909	0.0100±0.0030	1.1611±0.0987	0.4253±0.0021	0.6660±0.0275	0.3818±0.0048	0.5746±0.0154
f10	0.0000±0.0000	0.0000±0.0000	0.0000±0.0000	0.8656±0.0497	2.6121±0.2390	0.7051±0.0019	3.0466±0.1052	0.6639±0.0142	2.6850±0.0631
f11	0.0096±0.0166	0.0000±0.0000	0.0000±0.0000	0.0000±0.0000	0.0000±0.0000	0.0000±0.0000	0.0000±0.0000	0.0000±0.0000	0.0000±0.0000
c1	0.0000±0.0000	0.0000±0.0000	0.0000±0.0000	0.0000±0.0000	0.0000±0.0000	0.0000±0.0000	0.0000±0.0000	1.3477±0.0370	0.0000±0.0000

c2	0.0000±0.0000	0.0000±0.0000	0.0000±0.0000	0.0000±0.0000	0.0000±0.0000	1.0097±0.0002	0.0000±0.0000	1.2938±0.0162	1.2075±0.0269
c3	0.0000±0.0000	0.0000±0.0000	0.0000±0.0000	0.0000±0.0000	0.0000±0.0000	1.0883±0.0064	0.0000±0.0000	1.2130±0.0068	1.2812±0.2304
c4	0.0000±0.0000	0.0000±0.0000	0.0000±0.0000	0.0000±0.0000	0.0000±0.0000	1.6077±0.0505	1.6195±0.0728	2.2030±0.031	1.8734±0.2017
c5	0.0000±0.0000	0.0000±0.0000	0.0000±0.0000	0.0000±0.0000	0.0000±0.0000	0.0000±0.0000	0.0000±0.0000	1.2695±0.0075	1.2658±0.0429
c6	0.0000±0.0000	0.0000±0.0000	0.0000±0.0000	0.0000±0.0000	0.0000±0.0000	0.0000±0.0000	0.0000±0.0000	0.0000±0.0000	1.0339±0.0165
c7	3.0129±0.5396	2.4193±0.0888	2.5504±0.0427	2.0624±0.0249	1.9873±0.0158	1.0629±0.0085	1.4022±0.0125	1.1217±0.1141	1.1507±0.1679
c8	0.0000±0.0000	0.0000±0.0000	0.0000±0.0000	0.0000±0.0000	0.0000±0.0000	1.3732±0.0109	1.9673±0.2035	1.3389±0.0536	2.0835±0.1336
c9	0.0000±0.0000	0.0000±0.0000	0.0000±0.0000	0.0000±0.0000	0.0000±0.0000	0.0000±0.0000	0.0000±0.0000	0.0000±0.0000	1.1489±0.0309
c10	0.0000±0.0000	0.0000±0.0000	0.0000±0.0000	0.0000±0.0000	0.0000±0.0000	1.2349±0.0177	1.1792±0.0439	1.1487±0.0103	1.0821±0.0162
c11	0.0000±0.0000	0.0000±0.0000	0.0000±0.0000	0.0000±0.0000	0.0000±0.0000	1.1425±0.0128	1.2282±0.0239	1.0873±0.0079	1.1222±0.0154
c12	1.5268±0.1603	1.2443±0.012	1.2569±0.024	1.2169±0.1075	1.1595±0.0529	2.1033±0.0781	2.4593±0.0809	1.6878±0.0564	1.7227±0.1338

Supplementary Table S2 Identification of carotenoids

**Peak no.**	**Retention time (min)**	**$\lambda_{vis-max}$ (nm)**	**$\lambda_{vis-acyl}$ (nm)**	**$ESI^{-}MS^{-}$(*m/z*)**	**Aglycone**	**Main identified molecule**	**References**	**Standard**
c1	14.871	458	327	601, 583, 565, 491	Violaxanthin	(13*Z*)-Violaxanthin	[1]	/
c2	16.240	468	416	601, 583, 565, 491	Violaxanthin	(all-*E*)-Violaxanthin	[2]	/
c3	19.086	447	309	601, 583, 565	Luteoxanthin	(all-*E*)-Luteoxanthin	[3]	/
c4	22.965	464	327	601, 583, 565, 491	Violaxanthin	(9*Z*)-Violaxanthin	[1]	/
c5	25.475	468	333	569, 551, 534	Lutein	15/15' *Z*-Lutein	[4]	/
c6	26.861	466	330	569, 551, 534	Lutein	13/13' *Z*-Lutein	[1]	/
c7	27.094	473	445	569, 551, 534	Lutein	(all-*E*)-Lutein	[2]	(all-*E*)-Lutein
c8	31.584	476	423	569, 551, 534	Zeaxanthin	(all-*E*)-Zeaxanthin	[2]	(all-*E*)-Zeaxanthin
c9	33.430	473	417	569, 551	Cryptoxanthin	(all-*E*)-Cryptoxanthin 5,6-epoxide	[5]	/
c10	39.060	471	422	552, 534, 460, 442	Cryptoxanthin	β-Cryptoxanthin	[6]	/
c11	41.411	470	339	537, 445	β-Carotene	(13*Z*)-β-Carotene	[2]	/
c12	43.722	479	425	537, 445	β-Carotene	(all-*E*)-β-Carotene	[2]	(all-*E*)-β-Carotene

Supplemental Table S3 RNA sequencing data and corresponding quality control

Sample	Clean Reads	Raw Reads	Mapped Reads	Q30 (%)	Multiple_Mapped	Uniquely_Mapped
S1-1	36716138	40020440	30385839 (82.76%)	92.06	840616 (2.77%)	29545223 (97.23%)
S1-2	41725534	44889950	34680066 (83.11%)	92.35	969471 (2.80%)	33710595 (97.20%)
S1-3	40332226	43491580	33554818 (83.20%)	92.03	932170 (2.78%)	32622648 (97.22%)
S2-1B	40571680	43828036	33371370 (82.25%)	92.11	934708 (2.80%)	32436662 (97.20%)
S2-2B	40059776	43172046	32813282 (81.91%)	92.22	905679 (2.76%)	31907603 (97.24%)
S2-3B	41261098	44467414	33969549 (82.33%)	92.29	965600 (2.84%)	33003949 (97.16%)
S2-1NB	43862630	47357900	36163868 (82.45%)	92.7	1048862 (2.90%)	35115006 (97.10%)
S2-2NB	38994276	42559076	32065199 (82.23%)	92.2	937380 (2.92%)	31127819 (97.08%)
S2-3NB	39397158	43585876	32271557 (81.91%)	92.43	935569 (2.90%)	31335988 (97.10%)
S3-1B	40591598	44089542	33190087 (81.77%)	92.21	952495 (2.87%)	32237592 (97.13%)
S3-2B	37290232	40362254	30317837 (81.30%)	91.97	849138 (2.80%)	29468699 (97.20%)

S3-3B	37554048	40672938	30680578 (81.70%)	92.1	886493 (2.89%)	29794085 (97.11%)
S3-1NB	42554194	46011234	34755914 (81.67%)	92	1002209 (2.88%)	33753705 (97.12%)
S3-2NB	40506620	43947308	32990294 (81.44%)	92.17	955009 (2.89%)	32035285 (97.11%)
S3-3NB	40707728	43907936	33198098 (81.55%)	92.12	959258 (2.89%)	32238840 (97.11%)
S4-1B	37536640	40495652	30509418 (81.28%)	91.89	1083665 (3.55%)	29425753 (96.45%)
S4-2B	39478744	42433540	32183788 (81.52%)	92.29	1058494 (3.29%)	31125294 (96.71%)
S4-3B	39434174	42797342	32098868 (81.40%)	92.12	1140822 (3.55%)	30958046 (96.45%)
S4-1NB	38879760	42195930	31923458 (82.11%)	92.74	1384312 (4.34%)	30539146 (95.66%)
S4-2NB	38129282	41736630	31171270 (81.75%)	92.43	1333942 (4.28%)	29837328 (95.72%)
S4-3NB	38128806	43139826	31238241 (81.93%)	92.31	1299691 (4.16%)	29938550 (95.84%)
S5-1B	40906322	44263900	33486074 (81.86%)	92.01	1114657 (3.33%)	32371417 (96.67%)
S5-2B	39961288	42999608	33060094 (82.73%)	92.19	1208657 (3.66%)	31851437 (96.34%)
S5-3B	41175196	44448590	34236089 (83.15%)	92.47	1208457 (3.53%)	33027632 (96.47%)

S5-1NB	41332890	44561394	34027885 (82.33%)	91.03	1312733 (3.86%)	32715152 (96.14%)
S5-2NB	41877524	45137776	34885895 (83.30%)	92.37	1378360 (3.95%)	33507535 (96.05%)
S5-3NB	39139220	42082128	32504188 (83.05%)	92.39	1259624 (3.88%)	31244564 (96.12%)

Supplementary Table S4 Different blotch formation in different species

**Species**	**Cultivar**	**Tissue**	**Blotch color**	**Blotch pigments**	**Non-blotch color**	**Non-blotch pigments**	**Gene(s) correlating with blotch or non-blotch formation**	**References**
Senecio cruentus	JeCB	petal	white	no anthocyanin	carmine	two cyaniding derivatives and one delphinidin derivative	*ScCHS2*, *ScF3H1*, *ScDFR3*, *ScANS*, *ScbHLH17*	[7]
Senecio cruentus	JePB	petal	white	no anthocyanin	pink	three pelargonidin derivatives and one cyaniding derivative	*ScCHS2*, *ScF3H1*, *ScDFR3*, *ScANS*, *ScbHLH17*	[7]
Paeonia suffruticosa	Shima Nishiki	petal	red	Cy3G5G, Pn3G5G, Pg3G5G, Pn3G, Pg3G	white	Cy3G5G, Pn3G5G, Pg3G5G, Cy3G, Pn3G, Pg3G	*PsDFR*, *PsMYB*, *PsWD40*	[8]
Paeonia suffruticosa	Qing Hai Hu Yin Bo	petal	purple	Cy3G5G, Pn3G5G, Cy3G, Pn3G	white	no anthocyanin	*PsCHS*, *PsMYB12*, *PsbHLH*, *PsWD40*	[9]
Paeonia suffruticosa	Jinrong	petal	purple		white		*PsCHS*, *PsF3'H*, *PsDFR*, *PsANS*	[10]
Paeonia suffruticosa	High Noon	petal	red	Cy3G5G, Pn3G5G, Cy3G, 8 flavones and flavonols	yellow	no anthocyanin, 8 flavones and flavonols	*PsMYB30*, *PsANS*	[11]
Viola × *wittrockiana* Gams.	Mengdie	petal	cyanic	cyanidin and delphinidin	yellow	no anthocyanin	*VwF3'5'H*, *VwDFR*, *VwANS*	[12]
Clarkia gracilis		petal	dark reddish-purple	cyanidin and peonidin	pink	malvidin	*CgF3'H*, *CgDFR2*, *CgF3'5'H*, *CgDFR1*	[13]
Clarkia gracilis		petal	dark reddish-purple		pink		*CgMYB1* alleles	[14]

Clarkia gracilis		petal	dark reddish-purple		pink		*CgMYB1*, *CgMYB6*, *CgMYB11*, *CgMYB12*	[15]
Mimulus lewisii		petal	pink		white	apigenin-7-glucuronide	*MlFNS*	[16]
Mimulus lewisii		petal	pink	low concentration of anthocyanins	white		*MlROI1* (an *R3-MYB*)	[17]
Mimulus lewisii		petal	pink		white		*MlLAR1* (an *R2R3-MYB*), *MlFLS*	[18]
Mimulus lewisii		petal	pink		white		*RED TONGUE* (*RTO*, an *R3-MYB*), *NEGAN* (an *R2R3-MYB*)	[19]
Mimulus lewisii		petal	pink		white		*MlMYB5a* (an *R2R3-MYB*)	[20]
Mimulus jungermannioides		petal	white		yellow	6 carotenoids: antheraxanthin, violaxanthin, *cis*-violaxanthin, deepoxyneoxanthin, neoxanthin (*cis* or *trans*), mimulaxanthin		[21]
Lilium spp.	Tiny Padhye	tepal	purple	high content of anthocyanin	white	no anthocyanin	*LhCHS*, *LhCHI*, *LhF3H*, *LhF3'H*, *LhDFR*, *LhUFGT*, *Lh3RT*	[22]
Lilium spp.	Lollypop	tepal	pink		white		*LhMYB12*	[23]
Lilium spp.	Latvia	tepal	red		green		*LhMYB12-Lat*	[24]

Lilium spp.	Montreux	tepal	dark red		red		*LhMYB19Long*, *LhMYB19Short*	[25]
Arachis hypogaea		testa	red	higher content of cyanidin and delphinidin	white		*AhPAL*, *AhC4H*, *AhCHS*, *AhF3'H*	[26]
Trifolium spp.		leaf	red		green		*RED LEAF* (an *R2R3-MYB*)	[27]

Supplementary Table S5 Other DEGs identified in anthocyanin biosynthesis-related pathways

**Function**	**Gene name**	**Gene abbreviation**	**Positive No.**	**Negtive No.**
Auxin	Auxin response factor	*ARF*	1	0
Flower development	MADS-box transcription factor family protein	*MADS*	3	0
Cell differentiation and flower morphological development	C2H2-type zinc finger family protein	*C2H2*	1	0
Jasmonate	Jasmonate-zim-domain protein	*JAZ*	1	0
Cytokinin	GATA transcription factor	*GATA*	1	0
Calcium ion binding	Calcium-dependent lipid-binding (CaLB domain) family protein	*CaLB*	3	2
Calcium ion binding	Calcium-binding EF-hand family protein	*CBL*	0	1
Calcium ion binding	P3B-ATPase PH1	*PH1*	0	1
Calcium ion binding	two-pore channel	*TPC*	0	1
Calcium ion binding	Calcium-dependent phospholipid-binding Copine family protein	*Copine*	0	1
Calcium ion binding	Early-responsive to dehydration stress family protein	*ERD*	0	1
Calcium ion binding	C2 calcium/lipid-binding plant phosphoribosyltransferase family protein	*C2*	1	0
Stress responses	Glutathione S-transferase family protein	*GST*	1	0
Abiotic stress responses	Basic-leucine zipper transcription factor family protein	*bZIP*	0	1
Abiotic stress responses	Far-red impaired responsive 1 family protein	*FAR1*	0	1
Abiotic stress responses	heat shock transcription factor	*HSF*	0	1

Supplementary Table S6 Primers required for key gene qRT-PCR expression

Primer name	Sequence (5'-3')
tubulin-F	GATGCCACCGCTGATGAGGACG
tubulin-R	AGTTGTCCCCTCAACTCAACCAGC
RC1G0025000-F	TTCACCTCCTCAAAGATGTTCC
RC1G0025000-R	AGTGAGTTCCAGTCTGTGATGTTTA
RC1G0494500-F	TGGAAGACCAAGAAAGGAACTG
RC1G0494500-R	GCTGCCAACCTATCCCTCA
RC7G0563900-F	CTACAAATCCTCGCCGCTAA
RC7G0563900-R	CCATTTGCCGTTGTGCTG
RC7G0212200-F	GCACTCACCTTCATCCTCCA
RC7G0212200-R	CAAGGTGTCGCCGATGTG
RC6G0470600-F	AAGCAACTGGAGTGATGTCGA
RC6G0470600-R	CGAGAGTTGGGATAATGGTGAT
RC2G0136900-F	GTTGTCCATAGCCACATTCCA
RC2G0136900-R	CATCTCAGTGTAAGTGATTGGCTC
RC0G0031100-F	TCCTTCTCTGGAGGGACTTCTT
RC0G0031100-R	CCAATCCCAGGCTTTCAGA
RC5G0593900-F	ACCACCAAAAGGCAGCAAA
RC5G0593900-R	TCCACCCACAATGCGACA
RC7G0058400-F	ATCAGACATACCCAACCTCCC
RC7G0058400-R	GGAGCCTTTCGGGATGTG
RC1G0363600-F	TCAACCTCTCCACAGACACCA
RC1G0363600-R	CTTGATGGGTTTCTGTCCGTAT
RC0G0030000-F	TTGGGTCTCTTATGGAGGGTC

RC0G0030000-R	CCCTGGATTCCCACTGCT
RC2G0406300-F	TCCCAACAACACGCAACC
RC2G0406300-R	ATTGACTCGCTAGTGCTCGG
RC7G0019000-F	GCGTCGTTGCGGTAAGAG
RC7G0019000-R	CTATTAGCGACCACCTATTCCC
RC0G0179800-F	CTCAAGAGCGTGGTGTTCG
RC0G0179800-R	TTTCAGTGGTTCTGTTAGGCC
RC5G0166100-F	CCGAGCACAACCATCCCT
RC5G0166100-R	CCTTTGCTGGGCTATCTGG
RC1G0093800-F	GGCTCAGGACAAGGAGTGGT
RC1G0093800-R	TCAATGTCCTCTTGCTCCCA
RC2G0093100-F	CATCAACATGTAGATCATCGACC
RC2G0093100-R	CCAGGCAAAGGTCGGAAT
RC5G0279600-F	AGTCCAGTAACACGCCTTCG
RC5G0279600-R	TGGAAATGGAAGACTGTTGATG
RC2G0471400-F	ACCCTCCTCTACTCTGGCTCA
RC2G0471400-R	GCAAAGAACGGCGAAATG

References

1. Bakó E, Deli J, Tóth G. HPLC study on the carotenoid composition of *Calendula* products. J Biochem Biophys Methods. 2002;53:241-250.

2. Al-Yafeai A, Malarski A, and Böhm V. Characterization of carotenoids and vitamin E in *R- rugosa* and *R-canina*: comparative analysis. Food Chem. 2017;242:435-442.

3. Schex R, Lieb VM, Jiménez VM, Esquivel P, Schweiggert RM, Carle R, Steingass CB. HPLC-DAD-APCI/ESI-MS^n analysis of carotenoids and α-tocopherol in Costa Rican *Acrocomia aculeata* fruits of varying maturity stages. Food Res Int. 2018;105:645-653.

4. Mendes-Pinto MM., Ferreira ACS, Oliveira MBPP, Pinho PGD. Evaluation of some carotenoids in grapes by reversed- and normal-phase liquid chromatography: a qualitative analysis. J Agric Food Chem. 2004;52(10):3182-3188.

5. Wan HH, Yu C, Han Y, Guo XL, Ahmad S, Tang AY, Wang J, Cheng TR, Pan HT, Zhang QX. Flavonols and Carotenoids in Yellow Petals of Rose Cultivar (*Rosa* 'Sun City'): A Possible Rich Source of Bioactive Compounds. J Agric Food Chem. 2018;66(16):4171-4181.

6. Garzon GA, Narvaez-Cuenca CE, Kopec RE, Barry AM, Riedl KM, Schwartz SJ. Determination of carotenoids, total phenolic content, and antioxidant activity of araza (*Eugenia stipitata Mcvaugh*), an amazonian fruit. J Agric Food Chem. 2012;60:4709-4717.

7. Qi FT, Liu YT, Luo YL, Cui YM, Lu CF, Li H, Huang H, Dai SL. Functional analysis of the ScAG and ScAGL11 MADS-box transcription factors for anthocyanin biosynthesis and bicolour pattern formation in *Senecio cruentus* ray florets. Hortic Res. 2022;23: uhac071.

8. Zhang XP, Zhao LY, Xu ZD, Yu XN. Transcriptome sequencing of *Paeonia suffruticosa* 'Shima Nishiki' to identify differentially expressed genes mediating double-color formation. Plant Physiol Biochem. 2018;123: 114-124.

9. Gu Z, Zhu J, Hao Q, Yuan YW, Duan YW, Men SQ, Wang, QY, Hou QZ, Liu ZA, Shu QY, Wang LS. A novel R2R3-MYB transcription factor contributes to petal blotch formation by regulating organ-specific expression of *PsCHS* in tree peony (*Paeonia suffruticosa*). Plant Cell Physiol. 2019;60(3):599-611.

10. Zhang YZ, Cheng YW, Ya HY, Xu SZ, Han JM. Transcriptome sequencing of purple petal spot region in tree peony reveals differentially expressed anthocyanin structural genes. Front Plant Sci. 2015;4:964-973.

11. Luan YT, Tang YH, Wang X, Xu C, Tao J, Zhao DQ. Tree peony R2R3-MYB transcription factor *PsMYB30* promotes petal blotch formation by activating the transcription of anthocyanin synthase gene. Plant Cell Physiol. 2022;63(8):1101-1116.

12. Li Q, Wang J, Sun HY, Shang X. Flower color patterning in pansy (*Viola* × *wittrockiana* Gams.) is caused by the differential expression of three genes from the anthocyanin pathway in acyanic and cyanic flower areas. Plant Physiol Biochem. 2014;84:134-141.

13. Martins TR, Berg JJ, Blinka S, Rausher MD, Baum DA. Precise spatio-temporal regulation of the anthocyanin biosynthetic pathway leads to petal spot formation in *Clarkia gracilis* (Onagraceae). New Phytol. 2013;197:958-969.

14. Martins TR, Jiang P, Rausher MD. How petals change their spots: *cis*-regulatory re-wiring in *Clarkia* (Onagraceae). New Phytol. 2017;216:510-518.

15. Lin RC, Rausher MD. Ancient gene duplications, rather than polyploidization, facilitate diversification of petal pigmentation patterns in *Clarkia gracilis* (Onagraceae). Mol Biol Evol. 2021;12:5529-5538.

16. LaFountain AM, Chen WJ, Sun W, Chen SL, Frank HA, Ding BQ, Yuan YW. Molecular basis of overdominance at a flower color locus. G3 (Bethesda). 2017;7:3947-3954.

17. Yuan YW, Sagawa JM, Young RC, Christensen BJ, Bradshaw Jr. HD. Genetic dissection of a major anthocyanin QTL contributing to pollinator-mediated reproductive isolation between sister species of *Mimulus*. Genetics. 2013;194:255-263.

18. Yuan YW, Rebocho AB, Sagawa JM, Stanley LE, Bradshaw Jr. HD. Competition between anthocyanin and flavonol biosynthesis produces spatial pattern variation of floral pigments between *Mimulus* species. Proc Natl Acad Sci USA. 2016;113:2448-2453.

19. Ding BQ, Patterson EL, Holalu SV, Li JJ, Johnson GA, Stanley LE, Greenlee AB, Peng FE, Bradshaw Jr. HD, Blinov ML, Blackman BK, Yuan YW. Two MYB proteins in a self-organizing activator-inhibitor system produce spotted pigmentation patterns. Curr Biol. 2020;30:802-814.

20. Zheng XY, Om KZ, Stanton KA, Thomas D, Cheng PA, Eggert A, Simmons E, Yuan YW, Smith GDC, Puzey JR, Cooley AM. The regulatory network for petal anthocyanin pigmentation is shaped by the MYB5a/NEGAN transcription factor in *Mimulus*. Genetics. 2021;217:iyaa036.

21. LaFountain AM, Frank HA, Yuan YW. Carotenoid composition of the flowers of *Mimulus lewisii* and related species: implications regarding the prevalence and

origin of two unique, allenic pigments. Arch Biochem Biophys. 2015;573:32-39.

22. Xu LF, Yang PP, Feng YY, Xu H, Cao YW, Tang YC, Yuan SX, Liu XY, Ming J. Spatiotemporal transcriptome analysis provides insights into bicolor tepal development in *Lilium* “Tiny Padhye”. Front Plant Sci. 2017;8:398-414.

23. Suzuki K, Suzuki T, Nakatsuka T, Dohra H, Yamagishi M, Matsuyama K, Matsuura H. RNA-seq-based evaluation of bicolor tepal pigmentation in Asiatic hybrid lilies (*Lilium* spp.). BMC Genomics. 2016;17:611-630.

24. Yamagishi M, Toda S, Tasaki K. The novel allele of the *LhMYB12* gene is involved in splatter-type spot formation on the flower tepals of Asiatic hybrid lilies (*Lilium* spp.). New Phytol. 2014; 201:1009-1020.

25. Yamagishi M. Isolation and identification of MYB transcription factors (MYB19Long and MYB19Short) involved in raised spot anthocyanin pigmentation in lilies (*Lilium* spp.). J Plant Physiol. 2020;250:153164.

26. Hu MD, Li JW, Hou MY, Liu XQ, Cui SL, Yang XL, Liu LF, Jiang XX, Mu GJ. Transcriptomic and metabolomics joint analysis reveals distinct flavonoid biosynthesis regulation for variegated testa color development in peanut (*Arachis hypogaea* L.). Sci Rep. 2021;11:10721.

27. Albert NW, Griffiths AG, Cousins GR, Verry IM, Williams WM. Anthocyanin leaf markings are regulated by a family of *R2R3-MYB* genes in the genus *Trifolium*. New Phytol. 2015;205:882-893.
